# Integrated Aortic-Valve-And-Ascending-Aortic Replacement vs. Partial Replacement in Bicuspid Aortic Valve-Related Aortopathy

**DOI:** 10.3389/fcvm.2021.771346

**Published:** 2021-12-22

**Authors:** Mi Chen, Wangli Xu, Yan Ding, Honglei Zhao, Pei Wang, Bo Yang, Huanyu Qiao, Wei Zhang, Chenyang Zhou, Junnan Jia, Tao Bai, Jinrong Xue, Junming Zhu, Yongmin Liu, Weimin Li, Lizhong Sun

**Affiliations:** ^1^Department of Cardiac Surgery, Beijing Anzhen Hospital, Capital Medical University, Beijing, China; ^2^Department of Cardiac Surgery, University Hospital of Zurich, University of Zurich, Zurich, Switzerland; ^3^Center for Applied Statistics and School of Statistics, Renmin University of China, Beijing, China; ^4^National Tuberculosis (TB) Clinical Lab, Beijing Chest Hospital, Capital Medical University, Beijing, China

**Keywords:** bicuspid aortic valve, aortopathy, bicuspid aortic valve-related aortopathy, aneurysm, aortic dilatation

## Abstract

**Objective:** We sought to evaluate the outcomes of integrated aortic-valve and ascending-aortic replacement (IR) vs. partial replacement (PR) in patients with bicuspid aortic valve (BAV)-related aortopathy.

**Methods:** We compared long-term mortality, reoperation incidence, and the cumulative incidence of stroke, bleeding, significant native valve or prosthetic valve dysfunction, and the New York Heart Association (NYHA) functional classes II-IV between inverse probability-weighted cohorts of patients who underwent IR or PR for BAV-related aortopathy in a single center from 2002 to 2019. Patients were stratified into different aortic diameter groups (“valve type” vs. “aorta type”).

**Results:** Among patients with “valve type,” aortic valve replacement in patients with an aortic diameter > 40 mm was associated with significantly higher 10-year mortality than IR compared with diameter 35–40 mm [17.49 vs. 5.28% at 10 years; hazard ratio (HR), 3.22; 95% CI, 1.52 to 6.85; *p* = 0.002]. Among patients with “aorta type,” ascending aortic replacement in patients with an aortic diameter 52–60 mm was associated with significantly higher 10-year mortality than IR compared with diameter 45–52 mm (14.49 vs. 1.85% at 10 years; HR, 0.04; 95% CI, 1.06 to 85.24; *p* = 0.03).

**Conclusion:** The long-term mortality and reoperation benefit that were associated with IR, as compared with PR, minimizing to 40 mm of the aortic diameter among patients with “valve type” and minimizing to 52 mm of the aortic diameter among patients with “aorta type.”

**Trial Registration:** Treatment to Bicuspid Aortic Valve Related Aortopathy (BAVAo Registry): ChiCTR.org.cn no: ChiCTR2000039867.

## Introduction

Bicuspid aortic valve (BAV) disease is the most common congenital cardiac disorder, being present in 1–2% of the general population (1). Associated aortopathy, the dilatation of the aortic sinuses, and ascending aorta are present in ~20–40% of patients with BAV (2). Evidence of phenotypic heterogeneity of BAV and BAV aortopathy has emerged in the last decades. The classification of Sievers is most widely adopted to describe the morphology of BAV, namely the valvular phenotype (3). For aortopathy, the ascending phenotype vs. root phenotype has been proposed to require individualized surgical approaches (4, 5). Although evidence supporting treatment of BAV and aortopathy as separate entity has increased, data on the combined (valve and aorta) pathological phenotypes remain scarce. A comprehensive understanding of the interaction between morphologic features and functional characteristics of the BAV and aortopathy along with transvalvular hemodynamics is required. In particular, the 2 long-debated hypotheses with respect to the pathogenesis of BAV-related aortopathy—namely, the genetic and the hemodynamic theories—may contribute to differing causative factors. Previous data from mixed BAV cohorts resulted in a broad spectrum of surgical treatment methods being suggested, ranging from very conservative approaches to very aggressive recommendations (6). Currently, among patients with BAV with significant valve dysfunction, the practice guideline recommended cutoff for concomitant ascending aortic replacement is 45 mm (7, 8). However, there is a lack of evidence to clarify the need for concomitant aortic valve replacement among patients with dilated aorta, but without significant BAV dysfunction. As etiologic hypotheses based on the phenotypic heterogeneity of BAV and aortopathy continue to be discussed, specific surgical approaches and timing may be required. The aim of this study was to compare the perioperative and follow-up benefits and risks of integrated aortic-valve-and-ascending-aortic replacement (IR) vs. partial replacement (PR) for BAV-related aortopathy.

## Methods

### Study Design

**Figure 1:**
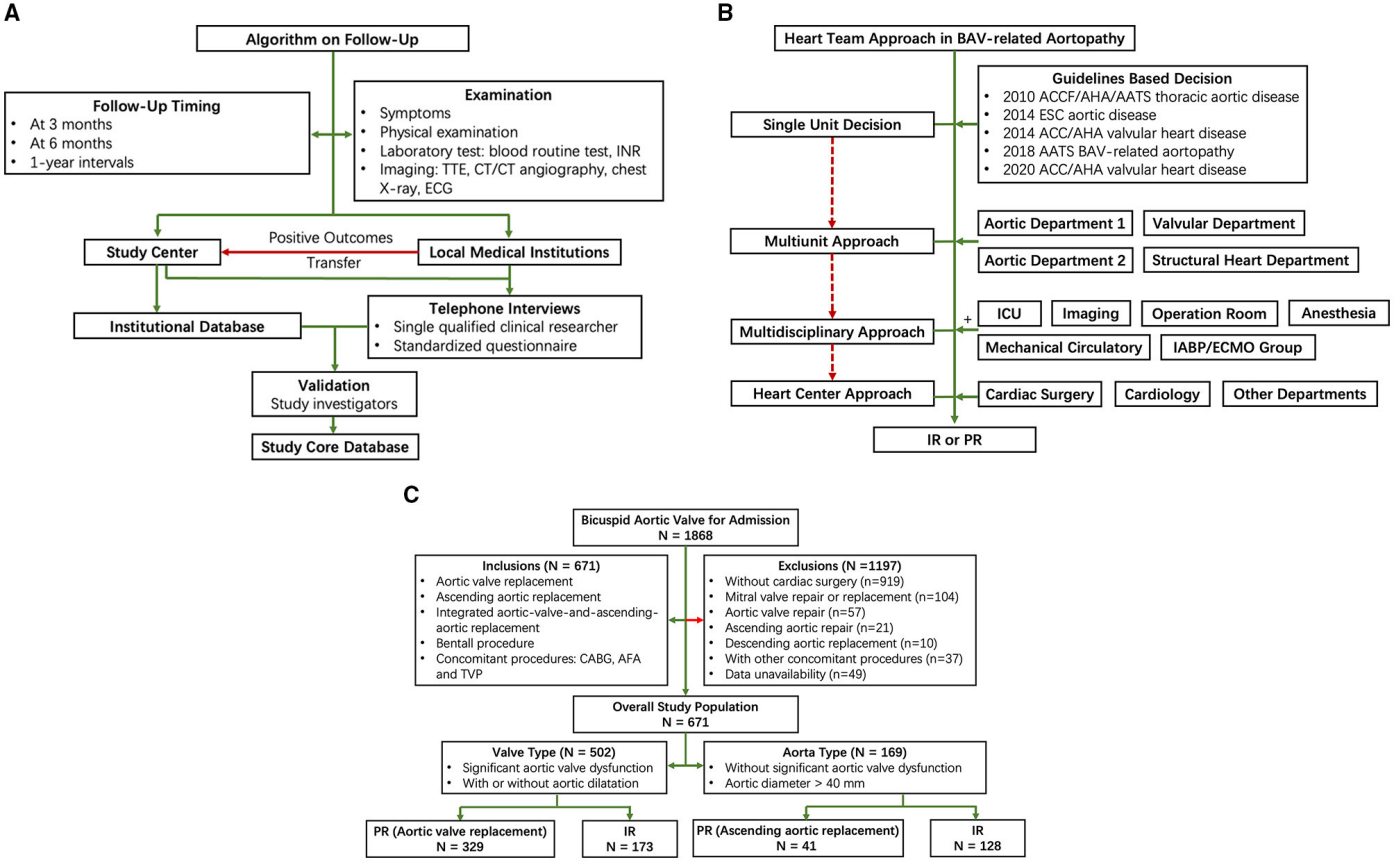
Flow diagram of study design. **(A)** Follow-up; **(B)** Heart team approach; and **(C)** Patient selection. AFA, atrial fibrillation ablation; CABG, coronary artery bypass graft; ICU, intensive care unit; INR, international normalized ratio; IR, integrated aortic-valve-and-ascending-aortic replacement; PR, partial replacement; TTE, transthoracic echocardiography; TVP, tricuspid valve plasty.

In this single-center inverse probability-weighted cohort study, we examined data from patients with BAV-related aortopathy who underwent IR or PR from January 1, 2002 to December 31, 2019 to evaluate the effect of surgical treatment on all-cause mortality and reoperation and the incidence of stroke, bleeding, significant native valve or prosthetic valve dysfunction, and the New York Heart Association (NYHA) functional class II-IV. This study was approved by an Institutional Review Board and the Institutional Review Board waived the need for a written informed consent of the patient. This study was registered with chictr.org.cn (ChiCTR2000039867, Methods in Supplementary Material). The patients were followed at 3 months, 6 months, and 1-year interval. Study investigators verified and validated investigation outcomes from the institutional database and standardized telephonic interviews (Figure 1A and Methods in Supplementary Material).

### Study Population and Pathophysiologic Classification

Patients were included in this study if they were diagnosed as BAV and underwent IR or PR. Decisions on IR or PR were based on the practical guidelines (6–10). Stepwise heart team approaches were taken by single unit (guidelines-based decision), multiunit approach, multidisciplinary approach, and heart center approach when symptoms and frailty, the burden of comorbidities, and technical aspects of patients necessitated further evaluation (Figure 1B). In particular, a risk factor of dissection (family history of aortic dissection, if the rate of increase in diameter is ≥ 0.3 cm per year or uncontrollable hypertension) and relatively low body surface area would be taken into consideration in the heart team to decide whether to perform a concomitant ascending aortic replacement; moderate aortic stenosis/aortic regurgitation (AS/AR), morphology of BAV, the diameter of the aorta, and the prognosis with untreated BAV would be taken into consideration in heart team to decide whether to perform a concomitant aortic valve replacement.

Based on emerging phenotypic heterogeneity of BAV-related aortopathy (3–5), we propose a simple nomenclature classification to include the valve and aorta together. We propose the terms “valve type” and “aorta type” to represent the most dysfunctional part among BAV-related aortopathy. Criteria for “valve type” included: (1) significant aortic valve dysfunction and (2) with or without aortic dilatation. Criteria for “aorta type” included: (1) without significant aortic valve dysfunction and (2) aortic diameter (aortic sinuses or ascending aorta) > 40 mm (Figure 1C).

Among patients with “valve type,” aortic valve replacement, as PR, was compared with IR. Among “aorta type,” ascending aortic replacement, as PR, was compared with IR. IR was defined as surgical treatment including aortic valve replacement and ascending aortic replacement.

Patients undergoing either the Bentall procedure (with coronary artery ostia reimplantation) or the Wheat procedure (11) (without coronary artery ostia reimplantation) were included as IR. Patients with concomitant coronary artery bypass graft (CABG), atrial fibrillation ablation (AFA), or tricuspid valve plasty (TVP) were included to improve statistical power. Exclusion criteria were concomitant mitral valve repair or replacement, aortic valve repair, ascending aortic repair, and descending aortic replacement, given that double valve replacement is associated with worse ventricular function and increased risk of bleeding.

### Echocardiographic Evaluation

Transthoracic echocardiography and transesophageal echography play key roles to screen pathophysiologic types. Function and morphology of BAV were verified and re-evaluated by the echocardiographic core laboratory based on the 2020 American College of Cardiology/American Heart Association (ACC/AHA) guideline (12). The criteria to evaluate the severity of aortic valve dysfunction were as follows: (1) severe AS was defined as aortic Vmax ≥ 4 m/s or mean ΔP ≥ 40 mm Hg; (2) moderate AS was defined as 20 mm Hg < mean ΔP <40 mm Hg; (3) severe AR was defined as vena contracta > 0.6 cm or effective regurgitant orifice (ERO) ≥ 0.3 cm^2^; (4) moderate AR was defined as 0.3 cm < vena contracta <0.6 cm; and (5) mild stenosis and regurgitation were regarded as normal valve function. Accordingly, severe AS or severe AR was regarded as significant aortic valve dysfunction. The remaining valve function was regarded as BAV without significant dysfunction. Based on the classification described by Sievers et al. (3), BAV morphology was classified into type 0 without raphe, type 1 with 1 raphe, and type 2 with 2 raphes (also called unicuspid aortic valve) according to the presence and number of raphes.

The aortic evaluation included the diameter of aortic sinuses and the ascending aorta by echocardiography. The aortic diameter was defined as the largest of the 2 diameters measured at the aortic sinus and the ascending aorta.

### Stratification Workflow

Stratification was based on the ascending aortic diameter. First, we stratified the study patients with 5-mm intervals roughly according to the current guidelines (9). For “valve type,” the stratification categories were: (1) 35–40 mm group and > 40 mm group and (2) 35–45 mm group and > 45 mm group. For “aorta type,” the stratification categories were: (1) 45–50 mm group and > 50 mm group and (2) 45–55 mm group and > 55 mm group.

Second, based on the results of the Cox proportional-hazards model, a 1-mm interval was taken to modify the trial categories. Equivalent dimension intervals were taken in two cohorts to achieve appropriate study power assessment if a 1-mm interval was needed.

### Study Endpoints

The primary endpoints were mortality and reoperation. Secondary endpoints included the cumulative incidence of stroke, bleeding, significant native valve or prosthetic valve dysfunction, and the NYHA functional classes II-IV. Safety endpoints included the freedom of cumulative incidence of death, reoperation for complications, non-elective cardiovascular surgery for adverse events, and deep wound infection within 1 month.

### Statistical Analysis

This study was designed to have a power of at least 90%, at an alpha level of 0.05, to detect a between-group hazard ratio of 3.5 for the analysis of mortality among patients with “valve type” with ascending aortic diameter of > 40 mm at 10 years and among patients with “aorta type” with ascending aortic diameter of > 55 mm at 10 years. Patients with smaller or larger diameters were included as contrast (13). We predicted IR that was associated with lower mortality in larger ascending aortic diameter and PR that was associated with lower mortality in smaller ascending aortic diameter.

We used inverse probability weighting to limit confounding by indication, particularly for the Sievers classification, valve function, and aortic phenotype (Methods section in the Supplementary Material). In each diameter group, non-parsimonious logistic regression was used to estimate probability of each patient to undergo IR or PR. Stabilized weights were calculated by dividing the marginal probability of the observed procedure by propensity score for the treatment received. The balance between the treatment groups was assessed with the use of standardized mean differences. A standardized mean difference of 10% or less was deemed to be the ideal balance and a standardized mean difference of 20% or less was deemed to be an acceptable balance.

The Cox proportional-hazards model with a robust variance estimator was used to compare long-term mortality between the groups. Separate analyses of the weighted population were adjusted for sinus diameter or included surgeon as a random effect. To address non-proportional hazards, the restricted mean survival time was estimated to describe the overall effect of treatment during the study period. Subdistribution hazards in the weighted populations were estimated with the method of Fine and Gray. SEs were estimated with the use of 500 bootstrap replicates.

To explore the diameter-dependent effect of different procedures on death and reoperation, the Cox proportional-hazards model was fit to the entire weighted study population with the use of an interaction term for aortic diameter and procedure. SEs were calculated from 1,000 bootstrap replicates. All the tests of treatment effect were two-tailed with an alpha threshold of 0.05. Statistical analyses were performed with the use of R software, version 4.0.3 (R Foundation) and data management was performed with the use of SPSS software, version 24 (SPSS Incorporation, Chicago, Illinois, USA). Additional details with respect to the statistical analysis are provided in the Methods section in the Supplementary Material.

## Results

Of 1,868 patients who were diagnosed as BAV for admission during the study period, 671 patients were eligible for inclusion in this study. A total of 502 patients and 169 patients were included in “valve type” and “aorta type,” respectively (Figure 1C). Among patients with “valve type,” the median follow-up was 4.92 years in the PR cohort and 4.75 years in the IR cohort. Among patients with “aorta type,” the median follow-up time was 3.33 years in the PR cohort and 4.58 years in the IR cohort.

### Clinical Characteristics of “Valve Type” and “Aorta Type”

Different aortic diameter distribution and the aortic valve function and morphology emerged in “valve type” vs. “aorta type” (Supplementary Figures 5, 6, Supplementary Tables).

In terms of valve function, among “valve type,” a similar percentage of severe AS and AR was showed (47.4 vs. 47.8%), while among “aorta type,” moderate stenosis had a higher incidence than moderate regurgitation (18.3 vs. 11.8%). With respect to valve morphology, Sievers' type 1 was dominant in both the “valve type” and “aorta type” (51.2 vs. 60.4%), whereas type 0 had a higher percentage in “valve type” and the distribution of the Sievers classification was significantly different (*p* = 0.03).

**Figure 2:**
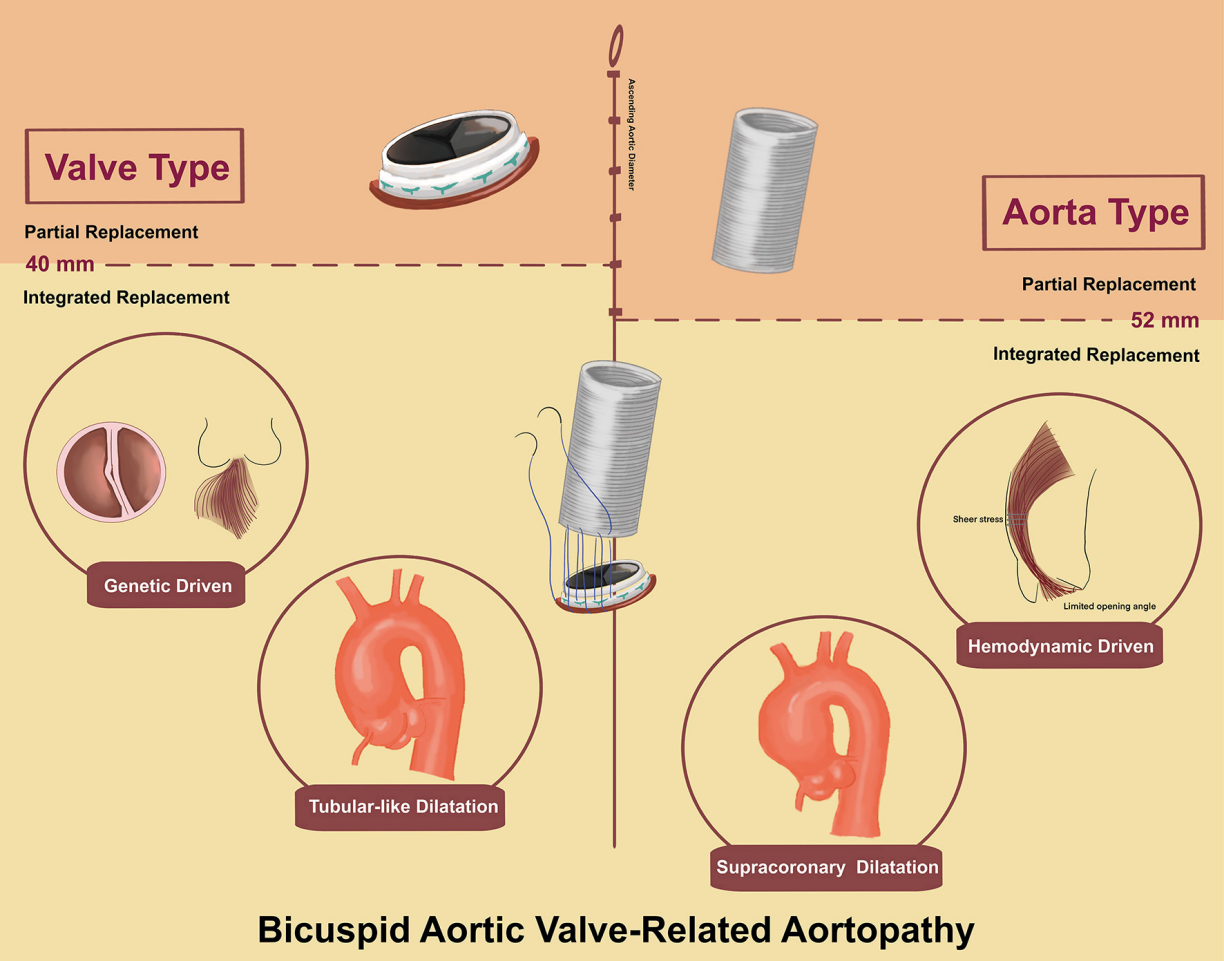
BAV-related Aortopathy.

Concerning the aortic dimensions, the “valve type” had an overall smaller dimension in aortic diameter (43.2 ± 8.2 vs. 53.6 ± 6.9 mm, *p* < 0.01), sinus diameter (35.6 ± 7.5 vs. 38.0 ± 7.7 mm, *p* < 0.01), and ascending aortic diameter (42.3 ± 8.4 vs. 52.9 ± 7.8 mm, *p* < 0.01). However, “aorta type” showed a higher ascending sinuses ratio than “valve type” (1.4 ± 0.3 vs. 1.2 ± 0.3 mm, *p* < 0.01), which was associated with supracoronary dilatation vs. tubular dilation (Figure 2).

### Inverse Probability Weighting Cohorts

Baseline and operative characteristics before inverse probability weighting are shown in Table 1. After using inverse probability weighting, the study population consisted of 333.3 vs. 178.7 in “valve type” and 47.4 vs. 132.4 in “aorta type,” which were not necessarily integers owing to inverse probability weighting. The standardized mean differences indicated an adequate match between PR and IR in both the types. Baseline characteristics of the cohorts were more balanced (standardized mean differences <15%) with considerable objectives reserved to enhance power (Supplementary Tables 3, 4).

**Table 1:** Baseline and operative characteristics before inverse probability weighting.

	**Valve type**			**Aorta type**		
**Characteristic**	**PR (*N* = 329)**	**IR (*N* = 173)**	**Effect size**	**PR (*N* = 41)**	**IR (*N* = 128)**	**Effect size**
Age (years)	48.7 ± 14.4	50.0 ± 12.1	−0.1	52.3 ± 9.0	50.4 ± 12.1	0.17
Year of surgery	2015 ± 3.1	2015.2 ± 3.1	−0.06	2016.4 ± 2.5	2015.2 ± 2.8	0.44
Study period			0.041			0.242
2002–2007	3 (0.9%)	1 (0.6%)		0	0	
2008–2013	94 (28.6%)	48 (27.7%)		7 (17.1%)	40 (31.2%)	
2014–2020	232 (70.5%)	124 (71.7%)		34 (82.9%)	88 (68.8%)	
Female sex			0.296			0.425
Male	228 (69.3%)	144 (83.2%)		25 (61.0%)	106 (82.8%)	
Female	101 (30.7%)	29 (16.8%)		16 (39.0%)	22 (17.2%)	
Valvular disease			0.204			0.556
Severe AS	166 (50.5%)	72 (41.6%)		-	-	
Severe AR	151 (45.9%)	89 (51.4%)		-	-	
Severe AS + AR	12 (3.6%)	12 (7.0%)		-	-	
Moderate AS	-	-		3 (7.3%)	34 (26.6%)	
Moderate AR	-	-		0	18 (14.1%)	
Moderate AS + AR	-	-		0	8 (6.3%)	
Mild or None AS/AR	-	-		38 (92.7%)	68 (53.1%)	
Severe AS						
Aortic V_max_ (m/s)	504.0 ± 76.1	500.4 ± 70.9	0.048	-	-	
Mean ΔP	63.3 ± 21.2	60.5 ± 19.0	0.137	-	-	
Severe AR						
ERO (mm^2^)	45.2 ± 15.7	52.4 ± 19.6	0.419	-	-	
Vena Contracta (mm)	7.6 ± 1.7	8.0 ± 1.2	0.258	-	-	
Severe AS +AR						
Aortic V_max_ (m/s)	504 ± 75.7	455.3 ± 41.5	0.737	-	-	
Mean ΔP (mm Hg)	70.1 ± 31.4	49.2 ± 7.2	0.81	-	-	
ERO (mm^2^)	43.0 ± 10.8	41 ± 13.5	0.169	-	-	
Vena Contracta (mm)	7.4 ± 0.9	7.4 ± 1.1	0			
Aortic valve diameter (mm)	23.3 ± 3.1	25.6 ± 4.0	0.668	22.7 ± 2.0	24.0 ± 2.4	−0.56
Aortic sinuses diameter (mm)	33.1 ± 5.4	40.3 ± 8.6	1.077	36.0 ± 4.4	38.6 ± 8.4	−0.339
Ascending aortic diameter (mm)	38.5 ± 5.8	49.6 ± 7.7	1.701	53.7 ± 4.5	52.7 ± 8.6	0.127
Aortic diameter (mm)^*^	39.2 ± 5.4	50.9 ± 7.2	1.922	53.7 ± 4.5	53.6 ± 7.5	0.014
Sievers's BAV type			0.159			0.014
Type 0	162 (49.2%)	71 (41.0%)		17 (41.5%)	50 (39.1%)	
Type 1	160 (48.6%)	97 (56.1%)		24 (58.5%)	78 (60.9%)	
Type 2	7 (2.1%)	5 (2.9%)		0	0	
Coexisting condition						
Hypertension	81 (24.6%)	57 (32.9%)	0.169	15 (36.6%)	47 (36.7%)	0
Diabetes mellitus	23 (7.0%)	14 (8.1%)	0.024	0	6 (4.7%)	0.143
Coronary artery disease	34 (10.3%)	15 (8.7%)	0.039	8 (19.5%)	17 (13.3%)	0.112
Peripheral vascular disease	7 (2.1%)	3 (1.7%)	0	1 (2.4%)	2 (1.6%)	0
Cerebrovascular disease	10 (3.0%)	5 (2.9%)	0	2 (4.9%)	6 (4.7%)	0.007
Congestive heart failure	159 (48.3%)	78 (45.1%)	0.053	12 (29.3%)	46 (35.9%)	0.091
Atrial fibrillation	9 (2.7%)	3 (1.7%)	0.035	1 (2.4%)	4 (3.1%)	0
COPD	2 (0.6%)	1 (0.6%)	0	0	0	
SBE	23 (7.0%)	2 (1.2%)	0.237	0	0	
Chronic kidney disease	1 (0.3%)	2 (1.2%)	0.051	0	3 (2.3%)	0.048
Renal dialysis	0	0		0	1 (0.8%)	0
Liver disease	8 (2.4%)	10 (5.8%)	0.149	3 (7.3%)	3 (2.3%)	0.156
Cancer	2 (0.6%)	0	0.025	2 (4.9%)	0	0.261
History of smoking	98 (29.8%)	58 (33.5%)	0.068	13 (31.7%)	51 (39.8%)	0.116
Dissection	1 (0.3%)	3 (1.7%)	0.106	0	7 (5.5%)	0.167
Obesity	15 (4.6%)	11 (6.4%)	0.058	2 (4.9%)	13 (10.2%)	0.111
Concomitant procedure						
CABG	18 (5.5%)	13 (7.5%)	0.063	2 (4.9%)	14 (10.9%)	0.131
TVP	0	0		0	0	
AFA	3 (0.9%)	1 (0.6%)	0	0	0	
Bentall procedure	0	129 (74.6%)		0	91 (71.1%)	
Prosthetic type			0.34			
Mechanical	271 (82.4%)	164 (94.8%)		-	122 (95.3%)	
Biological	58 (17.6%)	9 (5.2%)		-	6 (4.7%)	

* *Aortic diameter was defined as the maximum diameter between aortic sinuses and ascending aorta*.

*ΔP, pressure gradient between the LV and aorta; AFA, atrial fibrillation ablation; AR, aortic regurgitation; AS, aortic stenosis; BAV, bicuspid aortic valve; CABG, coronary artery bypass graft; COPD, chronic obstructive pulmonary disease; ERO, effective regurgitant orifice; IR, integrated aortic-valve-and-ascending-aortic replacement; PR, partial replacement; SBE, subacute bacterial endocarditis; TVP, tricuspid valve plasty; V_max_, maximum velocity*.

### Primary Endpoints in “Valve Type”

**Figure 3:**
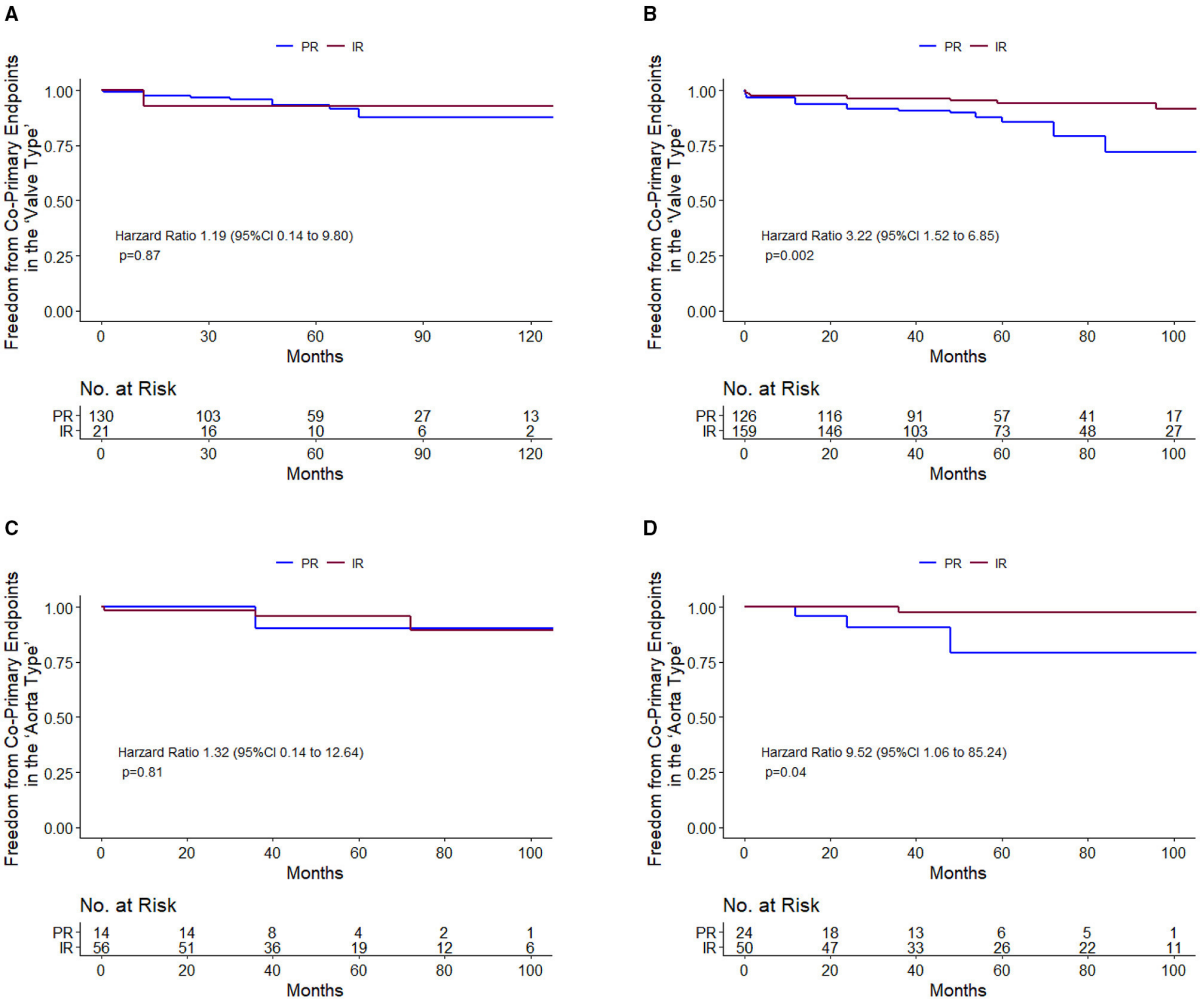
Primary endpoint survival curves for freedom from death and reoperation. 35–40 mm in valve type **(A)**; >40 mm in valve type **(B)**; 45–52 mm in aorta type **(C)**; and 52–60 mm in aorta type **(D)**.

**Figure 4:**
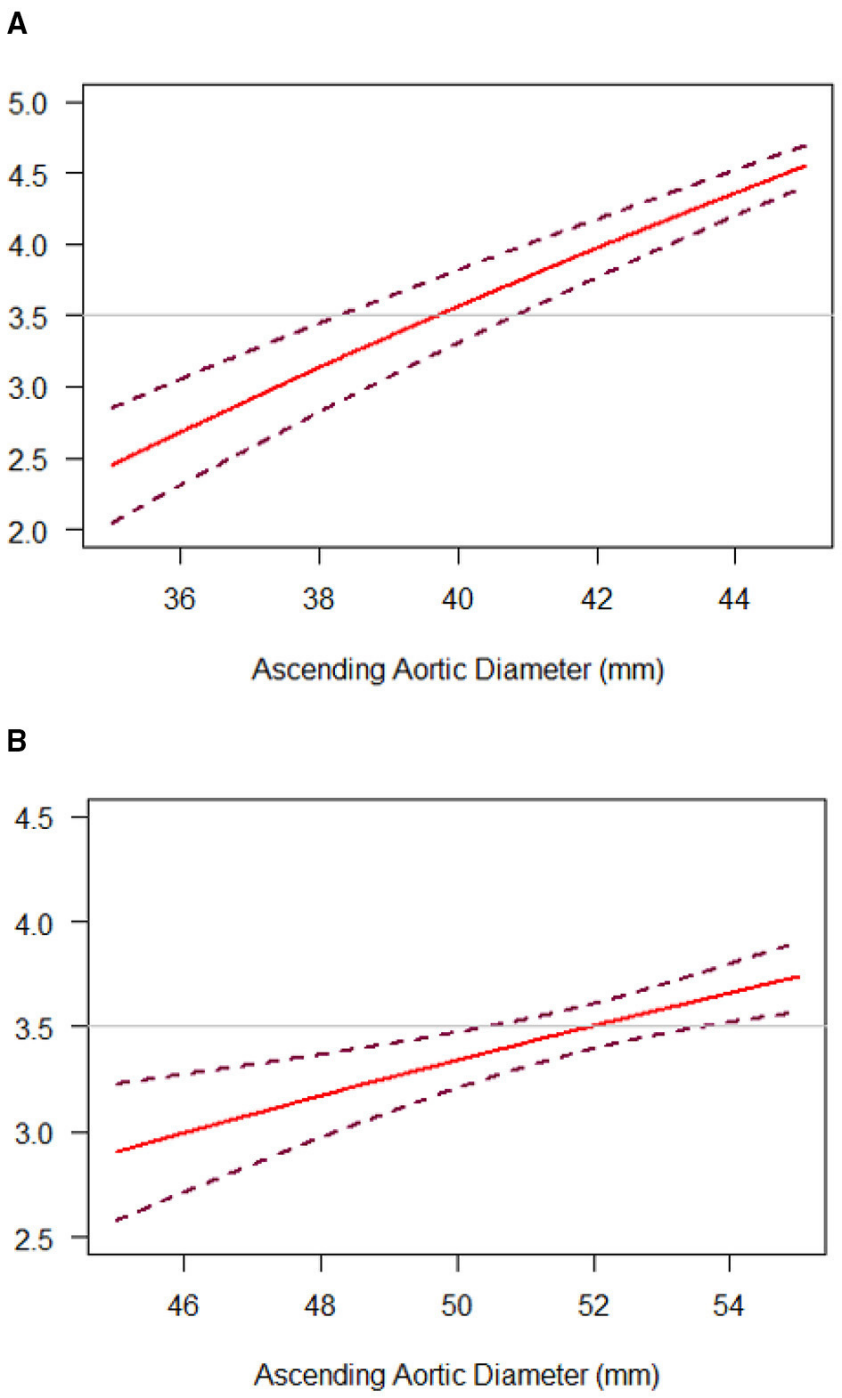
Diameter-dependent hazard of death and reoperation with IR, as compared with PR, in the “Valve Type” **(A)** or “Aorta Type” **(B)** groups. The hazard ratio (HR) for death and reoperation with IR, as compared with PR, was plotted against ascending aortic diameter as a continuous variable (solid lines). Dashed lines representing the 95% CI were obtained from bootstrap resampling. The horizontal line at 3.5 was consistent with reported HR.

Among diameter group of > 40 mm, IR was associated with a significantly lower cumulative incidence of all-cause mortality and reoperation than PR [11.98 vs. 4.65% at 5 years; 17.49 vs. 5.28% at 10 years; hazard ratio (HR), 3.22; 95% CI, 1.52 to 6.85; *p* = 0.002], but the difference was not significant among 35 to 40 mm diameter of the aorta (Table 2 and Figures 3A,B). These relationships were unaffected by multivariable adjustment or incorporation of the first operator as a random effect. Despite evidence of non-proportional hazards, the results of the comparisons of the restricted mean survival time (RMST) at 10 years were consistent with the marginal HRs but not at 5 years (Table 2). At 10-year RMST, PR gained−11.3 (95% CI,−19.6 to−3.0) additional months than IR (*p* = 0.007). Until 10 years, the ratio of life lost was 2.65 (1.3–5.6; *p* = 0.01) between PR and IR. When aortic diameter was examined as a continuous variable, the relative mortality benefits were associated with PR until ~40 mm of aortic diameter (Figure 4A). The individual endpoint of all-cause mortality was consistent with the co-endpoint, while reoperation showed no difference in IR vs. PR (Supplementary Material).

**Table 2:** Diameter-group differences in primary endpoints in “valve type*”.

**Variable**	**35–40 mm**			**>40 mm**		
	**PR (*N* = 130.1)**	**IR (*N* = 20.7)**	***p*-value**	**PR (*N* = 126.5)**	**IR (*N* = 158.9)**	***p*-value**
Hazard ratio (95% CI)						
Weighted PH model	1.19 (0.14–9.80)	Reference	0.87	3.22 (1.52–6.85)	Reference	0.002
Weighted PH model, with multivariable adjustment^†^	0.91 (0.14–5.86)	Reference	0.92	3.22 (1.51–6.84)	Reference	0.002
Weighted PH model, with surgeon as random effect	0.38 (0.10–1.42)	Reference	0.15	3.21 (1.49–6.89)	Reference	0.003
**5 years**						
Incidence (%)	5.48	7.04	0.78	11.98	4.65	0.02
RMST 5 years (95% CI)						
Difference (months)	0.95 (-4.96–6.85)	Reference	0.75	−2.15 (-5.18–0.88)	Reference	0.17
Ratio	1.02 (0.92–1.13)	Reference	0.76	0.96 (0.91–1.02)	Reference	0.17
Ratio of RMSL	0.68 (0.09–5.31)	Reference	0.72	1.96 (0.76–5.04)	Reference	0.16
**10 years**						
Incidence (%)	7.7%	7.04%	0.9	17.49%	5.28%	0.001
RMST (95% CI)^‡^						
Difference—months	−2.3 (-16.2–11.6)	Reference	0.74	−11.3 (-19.6−3.0)	Reference	0.007
Ratio	0.98 (0.87–1.11)	Reference	0.74	0.89 (0.83–0.97)	Reference	0.009
Ratio of RMSL	1.34(0.18–9.8)	Reference	0.77	2.65(1.3–5.6)	Reference	0.01

* *The overall numbers of patients in each group are not necessarily integers owing to inverse probability weighting*.

† *The analysis was adjusted for sinuses diameter*.

‡ *The RMST is the average duration of survival in a cohort over a prespecified follow-up period (5 and 10 years were reported here), as estimated by the area under the curve. The difference in the RMST is the average number of additional months gained in the treatment group (i.e., IR group minus PR group). The RMTL refers to the average number of days of life lost over a prespecified follow-up period; a ratio of more than 1.00 indicates that the treatment increased events incidence (or decreased the survival rate)*.

*IR, integrated aortic-valve-and-ascending-aortic replacement; RMST, restricted mean survival time; RMTL, restricted mean time lost; PH, proportional hazards; PR, partial replacement*.

### Secondary Endpoints in “Valve Type”

Among 35–40 mm of aortic diameter, the co-secondary endpoints occurred less frequently among PR than IR (HR, 0.39; 95% CI, 0.15 to 1.0; *p* = 0.05), but the difference was not significant among diameter > 40 mm. Concerning the freedom from the NYHA function classes II-IV, the difference was not significant in both the 35–40 mm group and > 40 mm group. Safety endpoints were not significantly different in PR vs. IR (Supplementary Material).

### Primary Endpoints in “Aorta Type”

Among diameter group of 52–60 mm, PR was associated with a significantly higher cumulative incidence of mortality and reoperation than IR (14.49 vs. 1.85% at 5 years; 14.49 vs. 1.85% at 10 years; HR, 9.52; 95% CI, 1.06 to 85.24; *p* = 0.04), but the difference was not significant among 45–52 mm diameter of the aorta (Table 3 and Figures 3C,D). These relationships were unaffected by multivariable adjustment or incorporation of the first operator as a random effect. The results of the comparisons of the restricted mean survival time were not significant at 5 and 10 years. When aortic diameter was examined as a continuous variable, the relative mortality benefits were associated with PR until ~52 mm of aortic diameter (Figure 4B). The individual endpoint of all-cause mortality was consistent with the co-endpoint, while reoperation showed no difference in IR vs. PR (Supplementary Material).

**Table 3:** Diameter-group differences in primary endpoints in “aorta type*.”

**Variable**	**45–52 mm**			**52–60 mm**		
	**PR (*N* = 15.7)**	**IR (*N* = 59.5)**	***p*-value**	**PR (*N* = 25.9)**	**IR (*N* = 51.5)**	***p*-value**
Hazard ratio (95% CI)						
Weighted PH model	1.32 (0.14–12.64)	Reference	0.81	9.52 (1.06–85.24)	Reference	0.04
Weighted PH model, with multivariable adjustment^†^	1.3 (0.15–11.44)	Reference	0.81	10.18 (1.32–78.76)	Reference	0.03
Weighted PH model, with surgeon as random effect	4.86 (0.41–57.54)	Reference	0.21	13.54 (1.12–163.26)	Reference	0.04
**5 years**						
Incidence (%)	6.93%	3.43%	0.56	14.49%	1.85%	0.03
RMST 5 years (95% CI)						
Difference (months)	−0.77 (-5.79–4.25)	Reference	0.76	−4.74 (-10.83–1.36)	Reference	0.13
Ratio	0.99 (0.9–1.08)	Reference	0.76	0.92 (0.82–1.03)	Reference	0.14
Ratio of RMSL	1.47 (0.14–15.22)	Reference	0.75	8.1 (0.88–74.97)	Reference	0.07
**10 years**						
Incidence (%)	6.93%	5.16%	0.79	14.49%	1.85%	0.03
RMST 10 year (95% CI)^‡^						
Difference—months	−6.97 (-19.89–5.96)	Reference	0.29	−14.37 (-32.2–3.46)	Reference	0.11
Ratio	0.94 (0.84–1.06)	Reference	0.3	0.88 (0.74–1.04)	Reference	0.14
Ratio of RMSL	2.63 (0.56–12.25)	Reference	0.22	7.16 (0.8–64.04)	Reference	0.08

* *The overall numbers of patients in each group are not necessarily integers owing to inverse probability weighting*.

† *The analysis was adjusted for sinuses diameter*.

‡ *The RMST is the average duration of survival in a cohort over a prespecified follow-up period (5 and 10 years were reported here), as estimated by the area under the curve. The difference in the RMST is the average number of additional months gained in the treatment group (i.e., IR group minus PR group). The RMTL refers to the average number of days of life lost over a prespecified follow-up period; a ratio of more than 1.00 indicates that the treatment increased events incidence (or decreased the survival rate)*.

*BAV, bicuspid aortic valve; IR, integrated aortic-valve-and-ascending-aortic replacement; RMST, restricted mean survival time; RMTL, restricted mean time lost; PH, proportional hazards; PR, partial replacement*.

### Secondary Endpoints in “Aorta Type”

The occurrence of co-secondary endpoints was not significantly different in the 45–52 mm group (HR, 2; 95% CI, 0.69–5.76; *p* = 0.2) and the 52–60 mm group (HR, 2.04; 95% CI, 0.76–5.46; *p* = 0.16). The NYHA functional class II-IV was lower among PR than IR (HR, 3.55; 95% CI, 0.99–12.72; *p* = 0.05) among the 52–60 mm group, whereas the difference was not significant among the 45–52 mm group.

Safety endpoints were shown no difference in PR vs. IR (Supplementary Material).

## Discussion

The main findings of this study are as follows: (1) A simple nomenclature classification can be used to describe the valve-and-aorta phenotype in BAV-related aortopathy; (2) IR was associated with long-term mortality and reoperation benefits compared with PR; and (3) The cutoff for IR was 45 mm in the “valve type” and 52 mm in the “aorta type.”

### Entity: Valve and Aorta

The prevalence of dilation of the ascending aorta among patients with BAV has been highly variable with reports ranging from 20 to 84% (14). Since the 1990s, these findings have generated two etiological hypotheses, “genetic” vs. “hemodynamic,” which remain debated. Supporters of the “genetic hypothesis” claim that a strong genetic role contributes to BAV-related aortopathy and more aggressive surgical intervention should be recommended, equivalent to Marfan syndrome (6, 15). Conversely, supporters of the “hemodynamic hypothesis” claim that abnormal flow patterns and asymmetrically increased wall stress resulting from BAV lead to proximal aortopathy and are less dangerous than described in the “genetic hypothesis” (16, 17). However, the marked heterogeneity of BAV-related aortopathy suggests more complex pathogenesis than simply “genetically determined” or “hemodynamically driven,” contributing to the increasing recognition of the entity of BAV and aortopathy complex.

### “Valve Type” and “Aorta Type”

The currently proposed phenotypes only focus on the valvular part (the Sievers classification) or aortic part (ascending phenotype vs. root phenotype) separately (4, 5). Based on the confirmed phenotype heterogeneity, we proposed a simple nomenclature, “valve type” and “aorta type,” in order to include valve and aorta equivalently. The proposed classification is easily instituted by the cardiologist and cardiac surgeon with high precision and generalizability. This classification not only assists in diagnosing BAV-related aortopathy as an entity, but may also improve preprocedural planning and provide long-term benefit. Using this classification may allow cardiac surgeons to follow a phenotype-determined intervention timing.

Notably, the high incidence of Sievers' type 0 and AR in “valve Type” was associated with more instinct abnormality in the light of “genetic hypothesis.” In contrast, “aorta type” with a supraannular dilation was associated with the “hemodynamic hypothesis,” which was consistent with Barker et al. (16) who reported BAV causing regional aortic wall shear stress due to abnormal BAV-related ascending aortic flow jet patterns. Therefore, our simple nomenclature may connect the two main hypotheses to clinical manifestation.

### Integrated Replacement vs. PR in the “Valve Type”

Several previous guidelines have addressed the management of BAV-related aortopathy from aggressive recommendations to a more conservative set of recommendations (6, 12). However, referred study concluded the cutoff based on isolated aortic valve replacement with different aortic dimensions, a lack of IR cohort in contrast (18). We compared our cohorts with IR vs. PR to evaluate both the long-term feasibility and perioperative safety. Optimally, surgery should be recommended as soon as the risk of watchful waiting exceeds the risk of surgical intervention. To conclude an effective cutoff of a mortality benefit, we chose a HR of 3.5 in our continuous variable line to predict the approximate aortic diameter, given that Michelena et al. (13) reported aorta diameter ≥ 40 mm was a predictor of aneurysm formation with a HR of 3.4. These study findings are also adopted generally in aortic aneurysm management.

### What Should be the Determinant for Integrated Replacement in the “Aorta Type”?

For “aorta type” without significant dysfunction, no related studies showed the optimal timing and indication for IR. Factors that need to be considered include aortic diameter, valve function, and presence of surgical risk factors. Given that calcific AS usually presents between the 5th and 7th decades (19), optimal timing for IR may be recommended even without significant aortic valve dysfunction. In this study, a supracoronary dilatation was shown in the “aorta type,” which demonstrated the dysfunction order. Based on the “hemodynamic hypothesis,” we assumed that the restricted opening angle of the BAV leaflets would result in more severe aortic wall shear stress. However, the related BAV function could be normal or only mildly dysfunctional and the evaluation of the precise opening angle of leaflets could be challenging, which means the BAV itself cannot adequately predict the prognosis. The dilated aorta, however, could play a role as an indicator of the harm dealt by the BAV, given that the abnormal BAV leaflets may continue dilating the native or artificial aorta without valve replacement. Therefore, rather than valve function or sinus diameter, we stratified the study patients according to ascending aortic diameter. A 52-mm was showed as the cutoff for IR, which was more aggressive than the current guideline recommendations for PR (55 or 50 mm in patients with risk factors); evidence for IR is lacking (7). However, such guideline recommendations are based on the observation that 60 mm represents a definite inflection point in the risk of aortic complications in both the BAV and tricuspid aortic valve (TAV), but with a lack of BAV-specific evidence to support this conclusion (20). It is worthwhile noting that operative risk plays a lesser role for experienced aortic surgeons nowadays. The aggressive treatment using advanced cardiac surgical techniques may show prophylactic benefit.

## Limitations

This study is limited by its retrospective and observational design. As numerous confounders exist in cardiac surgery studies, we used inverse probability weighting with well-balanced results to eliminate valve phenotype, aortic phenotype, and the other confirmed confounding factors between compared cohorts. Along with a limited study population, rather than propensity score pair matching, the use of inverse probability weighting reserved the maximal study population to enhance the generalizability and interpretability of study. Given a single-center study with a span of 20 years, we introduced the instrumental variables and selected “operator” as a strong variable to contrast the study outcomes. However, the utility of this classification and the timing of surgical intervention deserve future multicenter prospective trials.

## Conclusion

A simple classification, “valve type” and “aorta type,” could be used in BAV-related aortopathy to identify the surgical timing. In “valve type,” the long-term mortality benefit was associated with IR (valve and aortic replacement) when aortic diameter is larger than 40 mm, as compared with PR (valve replacement). In “aorta type,” the long-term mortality benefit was associated with IR (valve and aortic replacement) when aortic diameter is larger than 52 mm, as compared with PR (aortic replacement). The utility of this classification and the timing of surgical intervention deserve future international prospective trials to ensure unbiased race inclusion.

## Data Availability Statement

The original contributions presented in the study are included in the article/Supplementary Material, further inquiries can be directed to the corresponding author/s.

## Ethics Statement

The studies involving human participants were reviewed and approved by Beijing Anzhen Hospital Ethics Committee. Written informed consent for participation was not required for this study in accordance with the national legislation and the institutional requirements.

## Author Contributions

MC conceived and designed the study. WX, WL, and PW performed statistical design and analysis. MC, YD, HZ, BY, HQ, WZ, JX, and TB acquired the data. WL and JJ were in charge of the follow-up. MC and PW drafted the manuscript. LS and WL handled funding and supervision. LS, WL, WX, YL, and JZ made critical revision of the manuscript for key intellectual content. All authors contributed to the article and approved the submitted version.

## Funding

This study was supported by the Beijing Municipal Administration of Hospitals Clinical Medicine Development of Special Funding Support (No. ZYLX201503) and the Beijing Municipal Natural Science Foundation (No. 7202038).

## Conflict of Interest

The authors declare that the research was conducted in the absence of any commercial or financial relationships that could be construed as a potential conflict of interest.

## Publisher's Note

All claims expressed in this article are solely those of the authors and do not necessarily represent those of their affiliated organizations, or those of the publisher, the editors and the reviewers. Any product that may be evaluated in this article, or claim that may be made by its manufacturer, is not guaranteed or endorsed by the publisher.
