# Supplementary material for: Integrated Aortic-Valve-And-Ascending-Aortic Replacement vs. Partial Replacement in Bicuspid Aortic Valve-Related Aortopathy
Source: Front Cardiovasc Med. 2021 Dec 22;8:771346. doi: 10.3389/fcvm.2021.771346 (PMC8727738; doi:10.3389/fcvm.2021.771346)
Supplement: Supplementary file 1 [file Data_Sheet_1.docx]

**Integrated Aortic-Valve-and-Ascending-Aortic Replacement versus Partial Replacement in Bicuspid Aortic Valve-related Aortopathy**

**Supplementary Appendix**

Mi Chen, MD^1,2†^, Wangli Xu, PhD^3†^, Yan Ding, MD^1^, Honglei Zhao, MD, PhD^1^, Pei Wang^3^, Bo Yang, MD^1^, Huanyu Qiao, MD^1^, Wei Zhang, MD^1^, Chenyang Zhou, MD, PhD^1^, Junnan Jia^4^, Tao Bai, MD^1^, Jinrong Xue, MD, PhD^1,^ Junming Zhu, MD, PhD^1^, Yongmin Liu, MD, PhD^1^, Weimin Li, PhD^4^*, Lizhong Sun, MD, PhD^1^*

| **Table of Contents** | | **Page Number** |
| --- | --- | --- |
| Methods | Author Contributions | 6 |
| Methods | Funding Sources | 6 |
| Methods | Clinical Trial Information | 6 |
| Methods | Follow-up | 6 |
| Methods | Use of Inverse Probability Weighting | 7 |
| Methods | Use of Instrumental Variables | 8 |
| Methods | Restricted Mean Survival Time | 9 |
| Methods | Bootstrap Resampling | 10 |
| Methods | Power Calculations | 11 |
| Methods | Statistical Packages | 11 |
| Figure S1 | ROC Curve for Logistic Model of ‘Valve Type’ | 13 |
| Figure S2 | ROC Curve for Logistic Model of ‘Aorta Type’ | 14 |
| Figure S3 | Distribution of Stabilized Weights, Stratified by Diameter Group and Procedure for ‘Valve Type’ | 15 |
| Figure S4 | Distribution of Stabilized Weights, Stratified by Diameter Group and Procedure for ‘Aorta Type’ | 16 |
| Figure S5 | Aortic Dimension Distribution in “Valve Type” and “Aorta Type” | 17 |
| Figure S6 | Aortic Valve Function and Morphology in “Valve Type” and “Aorta Type” | 18 |
| Figure S7 | Kaplan-Meier Freedom from All-cause Mortality in the ‘Valve Type’, Stratified by Diameter and Procedures | 19 |
| Figure S8 | Kaplan-Meier Freedom from Cardiac Reoperation in the ‘Valve Type’, Stratified by Diameter and Procedures | 20 |
| Figure S9 | Kaplan-Meier Freedom from All-cause Mortality in the ‘Aorta Type’, Stratified by Diameter and Procedures | 21 |
| Figure S10 | Kaplan-Meier Freedom from Cardiac Reoperation in the ‘Aorta Type’, Stratified by Diameter and Procedures | 22 |
| Figure S11 | Kaplan-Meier Freedom from Cumulative Incidence of Stroke, Bleeding, NYHA function class II-IV in the ‘Valve Type’, Stratified by Diameter and Procedures | 23 |
| Figure S12 | Kaplan-Meier Freedom from NYHA function class II-IV in the ‘Valve Type’, Stratified by Diameter and Procedures | 24 |
| Figure S13 | Kaplan-Meier Freedom from Cumulative Incidence of Stroke, Bleeding, NYHA function class II-IV in the ‘Aorta Type’, Stratified by Diameter and Procedures | 25 |
| Figure S14 | Kaplan-Meier Freedom from NYHA function class II-IV in the ‘Aorta Type’, Stratified by Diameter and Procedures | 26 |
| Figure S15 | Kaplan-Meier Freedom from Safety Endpoints in the ‘Valve Type’, Stratified by Diameter and Procedures | 27 |
| Figure S16 | Kaplan-Meier Freedom from Safety Endpoints in the ‘Aorta Type’, Stratified by Diameter and Procedures | 28 |
| Table S1 | Models for Estimating Propensity Scores for ‘Valve Type’ | 29 |
| Table S2 | Models for Estimating Propensity Scores for ‘Aorta Type’ | 30 |
| Table S3 | Baseline and Operative Characteristics of the ‘Valve Type’ Before and After Inverse Probability Weighting | 31 |
| Table S4 | Baseline and Operative Characteristics of the ‘Aorta Type’ Before and After Inverse Probability Weighting | 33 |
| Table S5 | Aortic Diameter Distribution and Aortic Valve Function between the ‘Valve Type’ and the ‘Aorta Type’ | 35 |
| Table S6 | Diameter-Group (35-40 mm and >40 mm) Differences in Primary Endpoints in ‘Valve Type’ | 36 |
| Table S7 | Diameter-Group (35-45 mm and >45 mm) Differences in Primary Endpoints in ‘Valve Type’ | 38 |
| Table S8 | Diameter-Group (45-50 mm and >50 mm) Differences in Primary Endpoints in ‘Aorta Type’ | 40 |
| Table S9 | Diameter-Group (45-55 mm and >55 mm) Differences in Primary Endpoints in ‘Aorta Type’ | 42 |
| Table S10 | 5-year Subdistribution Hazard of Mortality and Reoperation among PR versus IR in the ‘Valve Type’ | 44 |
| Table S11 | 10-year Subdistribution Hazard of Mortality and Reoperation among PR versus IR in the ‘Valve Type’ | 45 |
| Table S12 | 5-year Subdistribution Hazard of Mortality and Reoperation among PR versus IR in the ‘Aorta Type’ | 46 |
| Table S13 | 10-year Subdistribution Hazard of Mortality and Reoperation among PR versus IR in the ‘Aorta Type’ | 47 |
| Table S14 | 5-year Subdistribution Hazard of Stroke, Bleeding, NYHA Function Class II-IV, or Significant Prosthetic Valve Dysfunction among PR versus IR in the ‘Valve Type’ | 48 |
| Table S15 | 10-year Subdistribution Hazard of Stroke, Bleeding, NYHA Function Class II-IV, or Significant Prosthetic Valve Dysfunction among PR versus IR in the ‘Valve Type’ | 49 |
| Table S16 | 5-year Subdistribution Hazard of Stroke, Bleeding, NYHA Function Class II-IV, or Significant Aortic Valve Dysfunction among PR versus IR in the ‘Aorta Type’ | 50 |
| Table S17 | 10-year Subdistribution Hazard of Stroke, Bleeding, NYHA Function Class II-IV, or Significant Aortic Valve Dysfunction among PR versus IR in the ‘Aorta Type’ | 52 |
| Table S18 | Between-Group 30-Day Freedom of the Cumulative Incidence of Death, Reoperation for Complications, Nonelective Cardiovascular Surgery for Adverse Events and Deep Wound Infection for PR and IR | 54 |

**METHODS**

**Author Contributions**

Mi Chen conceived and designed the study; Wangli Xu, Weimin Li and Pei Wang performed statistical design and analysis; Mi Chen, Yan Ding, Honglei Zhao, Bo Yang, Huanyu Qiao, Wei Zhang, Jinrong Xue, Tao Bai acquired the data; Weimin Li and Junnan Jia were in charge of the follow-up; Mi Chen and Pei Wang drafted the manuscript. Lizhong Sun and Weimin Li handled funding and supervision; Lizhong Sun, Weimin Li, Wangli Xu, Yongmin Liu and Junming Zhu made critical revision of the manuscript for key intellectual content.

**Funding Sources**

This study was supported in part by the Beijing municipal administration of hospitals clinical medicine development of special funding support (No. ZYLX201503) and Beijing municipal natural science foundation (No. 7202038).

**Clinical Trial Information**

This study was registered with chictr.org.cn (ChiCTR2000039867). The study was approved at institutional review board at Dec. 31^st^, 2019.

**Follow-up**

The patients were followed at 3, 6 months, and 1-year intervals. Patients were examined in the study center or local medical institutions. Standardized follow-up included symptoms, physical examination, laboratory tests (blood routine test, INR for mechanical aortic valve replacement), imaging (TTE, CT/CT angiography if applicable, chest X-ray, ECG). Patients with positive outcomes examined in local medical institutions were required callbacks and transferring to the study center for further examination. Institutional database was verified and validated by study investigators. Additionally, study participants (or relatives) were evaluated by telephone interview by a single qualified clinical researcher using a standardized questionnaire. Validated investigation outcomes were entered into study core database.

**Statistical Analysis**

Use of Inverse Probability Weighting

A frequently employed technique in clinical research is propensity score pair matching,

which creates matches between two patients who received different treatments but that had a similar probability of receiving the same treatment. In this design, many patients are excluded due to lack of an appropriate match. The generalizability and interpretability of results can be limited when patients are excluded in this manner. Our goal was to investigate the effect of treating all patients with partial replacement (average treatment effect); this is the effect estimate of a change in guidelines. Two techniques for the estimation of the average treatment effect were considered: inverse probability weighting and full matching. Inverse probability weighting incorporates the entire study population and weights patients by the inverse of the probability of receiving the observed treatment to create balanced pseudo-populations. Like inverse probability weighting, full matching incorporates the entire study population but does so by creating matched sets of variable size. This is in contrast to the more typical pair matching technique, which limits matched sets to 2 observations. Full matching is rarely used, and its use has primarily been restricted to binary or continuous outcomes. Therefore, we chose to use inverse probability weighting to address measured confounding.

Use of Instrumental Variables

The instrumental variable method was developed to control for unmeasured confounders. An instrumental variable method exploits a variable that influences treatment assignment with the following two conditions: it is independent of unmeasured confounders, and it does not directly affect outcome except through its influence on treatment assignment. For this analysis, we explored the possibility of using specific surgeon as a preference-based instrumental variable. Preference-based instrumental variables are particularly well-suited for observational studies of surgical treatments because: 1) the specific treatment a patient receives is often determined by the preferences of the surgeon performing the operation, and 2) for routine operations, patients are typically referred to surgeons in a manner that is not informed by severity or complexity of illness. In the case of valve replacement, a patient who received a partial replacement (PR) might have received an integrated aortic-valve-and-ascending-aortic replacement (IR) had been referred to a different surgeon. For this reason, the individual surgeon is theoretically a strong preference-based instrument in our study population.

Restricted Mean Survival Time

Time-to-event analyses typically employ Cox proportional hazards models. When comparing two groups in this fashion, an assumption is made that the ratio of the two hazard functions for the groups remains constant over time (e.g. proportional hazards). The Cox model is then used to estimate the unknown constant hazard ratio parameter. When there is concern, or evidence, of non-proportional hazards (e.g. the hazard ratio changes over time), the estimated hazard ratio may not be a meaningful measure of the between-group survival difference. Despite this issue, the Cochrane handbook instructs investigators to report hazard ratios to quantify between-group differences in survival, a recommendation supported by the CONSORT guidelines. For this reason, we report hazard ratios in our manuscript, but we also chose to report the restricted mean survival time (RMST). The RMST is the population’s average duration of event-free survival experienced over a pre-specified follow-up period and is calculated as the area under the survival curve. In our study, we chose 5 years and 10 years as our follow-up duration and made some comparation. A benefit of the RMST is that it does not rely on the proportional hazard assumption. Once the area under the survival curve is calculated, the difference in RMST between groups and the ratio of the two numbers provide useful estimates of the treatment effect on overall survival. The 5-year and 10-year RMST difference is interpreted as the average number of additional days gained by the treatment arm. The RMST ratio quantifies the percent of life gained by the treatment arm compared to the control arm. Similarly, the area above the survival curve corresponds to the number of days of life lost until the designated time point (e.g 5 years or 10 years), and is known as the restricted mean time lost. These model-free estimates of survival benefit also may have better power to detect between-group survival differences compared with the log-rank test in the setting of non-proportional hazards.

Bootstrap Resampling

The bootstrap is a resampling method which is commonly used to estimate confidence intervals for statistical inference. The main idea of bootstrap method is to conduct computations on the data itself to estimate specific statistics that are themselves calculated by the same data. Which means, the data is ‘pulling itself up by its own bootstrap.’ This procedure involves selecting multiple random samples with replacement from the original dataset and analyzing every sample through the same model. Resampling with replacement means that each observation is chose separately at random from the dataset. So a particular observation from the original data set may appear several times in a given bootstrap sample. The sample size of each bootstrap sample is equal to the size of the original data set. In our research, we use this method to find the relationship and variation trend between hazard ratios and the ascending aortic diameters. Bootstrap resampling is conducted in each aortic diameter group and the hazard ratio is computed by Cox regression model from each sample. Then the hazard ratio among recipients of partial replacement (PR) compared with recipients of integrated aortic-valve-and-ascending-aortic replacement (IR) can be plotted against ascending aortic diameters as a continuous variable, and we can determine a threshold value of ascending aortic diameter with a particular hazard ratio.

Power Calculations

The study was designed to achieve a power of more than 99% with an alpha-level of 0.05 to detect a between-group hazard ratio (HR) of 3.5 for 10-year cumulative incidence in patients with >40 mm of the ascending aortic diameter in the ‘Valve Type’, and in patients with 52-to-60 mm of the ascending aortic diameter in the ‘Aortic Type’. We assumed a ratio of 1:1 PR to IR, a median survival of 20 years, and an 18-year accrual period with no additional follow-up. Among patients with 35-to-40 mm of the ascending aortic diameter in the ‘Valve Type’, we had 99% power to detect a hazard ratio of 1.5 (assuming a ratio of 5:1 PR to IR). Among patients with 45-to-52 mm of the ascending aortic diameter in the ‘Aortic Type’, we had 98% power to detect a hazard ratio of 1.5 (assuming a ratio of 1:1 PR to IR).

Statistical Packages

Data was prepared for analysis in SPSS and statistical analyses were performed in R version 4.0.3. The *esc* package was used to compare baseline characteristics, 30-day mortality, and other perioperative outcomes. All-cause mortality and competing risks analyses were performed with the *survival* package and *survminer* package. Weighted Cox proportional hazards regression, with or without baseline characteristics forced into the model, was used with an independence covariance matrix and robust variance estimator to estimate the marginal and conditional effects of valve type on all-cause mortality. An independence working correlation matrix is recommended in the setting of inverse probability weighting and is employed in the *survival* package. We also used the *coxme* package to perform mixed-effects Cox proportional hazards models of the weighted pseudo-population with surgeon as a random baseline hazard. RMST differences, RMST ratios, and RMTL ratios were calculated and compared between groups with the *survRM2* package. The *fmsb* package is used to compute the odds ratio and the *pwr* package is used to complete the power calculation. Besides, the bootstrap process is achieved by *boot* package. In an effort to improve transparency in clinical research, the authors are willing to provide the code used to perform all calculations within the manuscript on request.

**Figure S1. ROC Curve for Logistic Model of ‘Valve Type’**


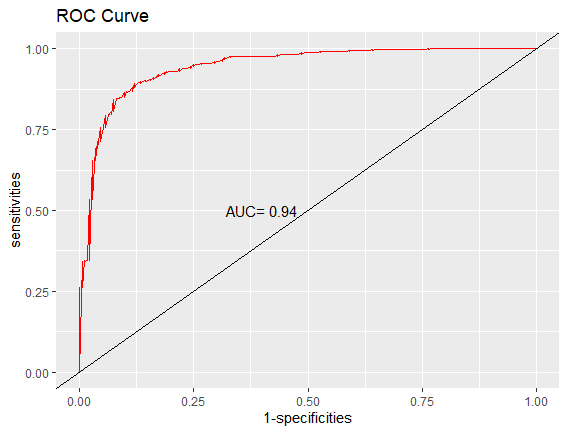


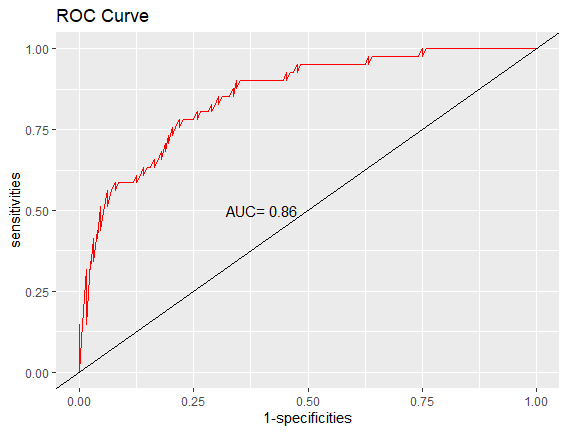
**Figure S2. ROC Curve for Logistic Model of ‘Aorta Type’**

**Figure S3. Distribution of Stabilized Weights, Stratified by Diameter Group and Procedure for ‘Valve Type’**


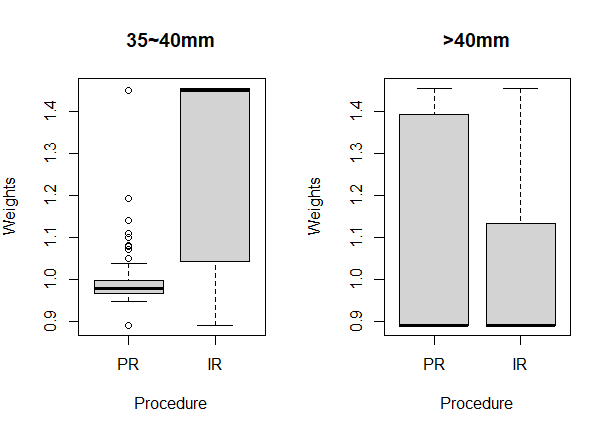


| PR: median 0.98 (IQR: 0.97 to 1.0)，range: 0.89 to 1.45 | PR: median 0.89 (IQR: 0.89 to 1.39)，range: 0.89 to 1.46 |
| --- | --- |
| IR: median 1.45 (IQR: 1.08 to 1.46)，range: 0.89 to 1.46 | IR: median 0.89 (IQR: 0.89 to 1.13)，range: 0.89 to 1.46 |

**Figure S4. Distribution of Stabilized Weights, Stratified by Diameter Group and Procedure for ‘Aorta Type’**


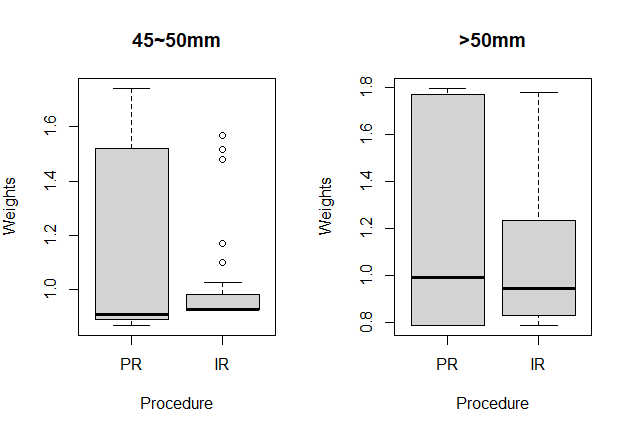


| PR: median 0.91 (IQR: 0.90 to 1.46)，range: 0.87 to 1.74 | PR: median 0.99 (IQR: 0.79 to 1.77)，range: 0.79 to 1.8 |
| --- | --- |
| IR: median 0.93 (IQR: 0.93 to 0.98)，range: 0.93 to 1.57 | IR: median 0.94 (IQR: 0.83 to 1.23)，range: 0.79 to 1.78 |

**Figure S5. Aortic Diameter Distribution and Aortic Valve Function between the ‘Valve Type’ and the ‘Aorta Type’**

**

**

**Figure S6. Aortic Valve Function and Morphology in “Valve Type” and “Aorta Type”**

**
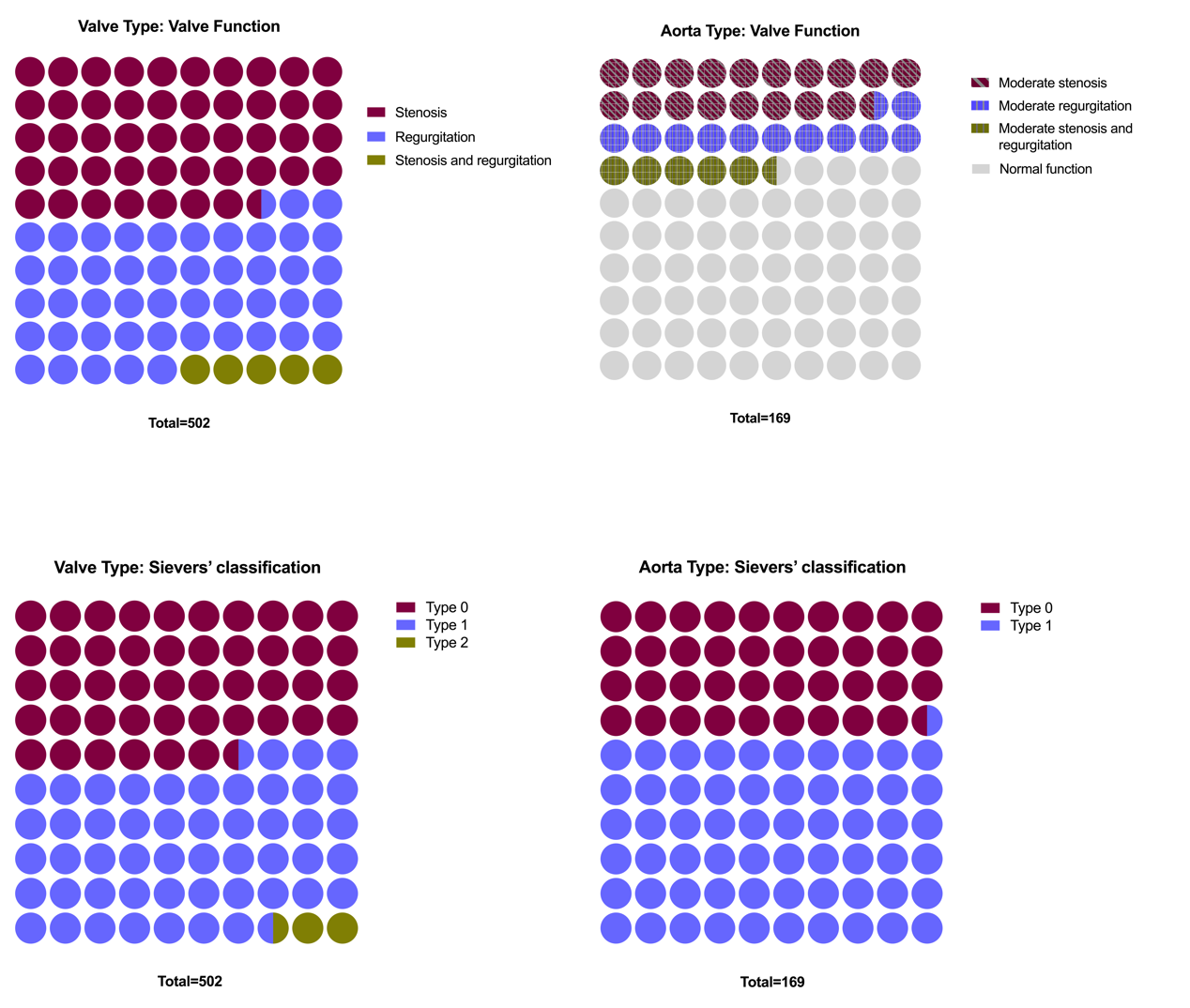
**

**Figure S7. Kaplan-Meier Freedom from All-cause Mortality in the ‘Valve Type’, Stratified by Diameter and Procedures**


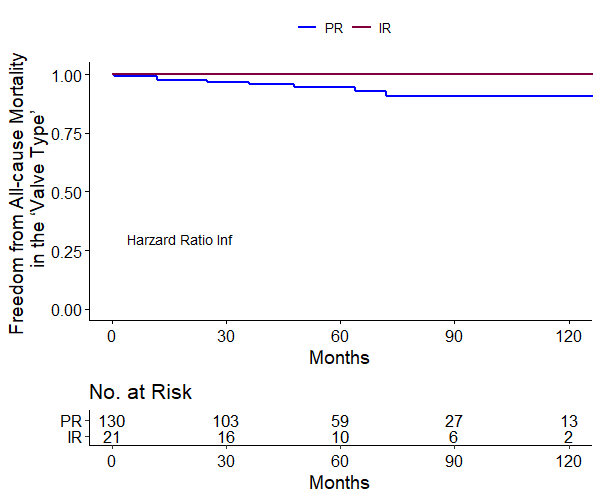
**S5-1 (35~40mm)**


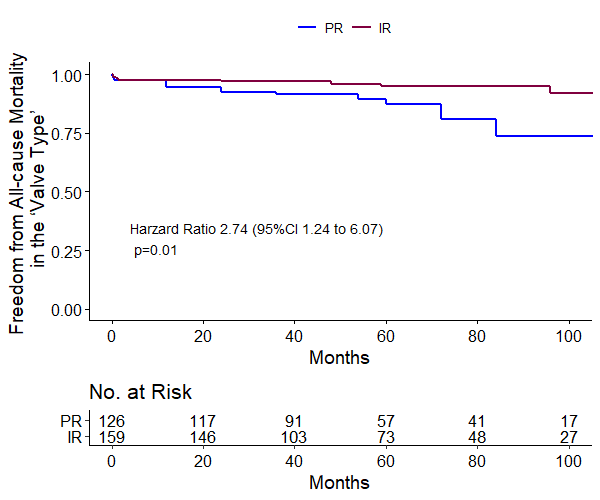
**S5-2 (>40mm)**

**Figure S8. Kaplan-Meier Freedom from Cardiac Reoperation in the ‘Valve Type’, Stratified by Diameter and Procedures**


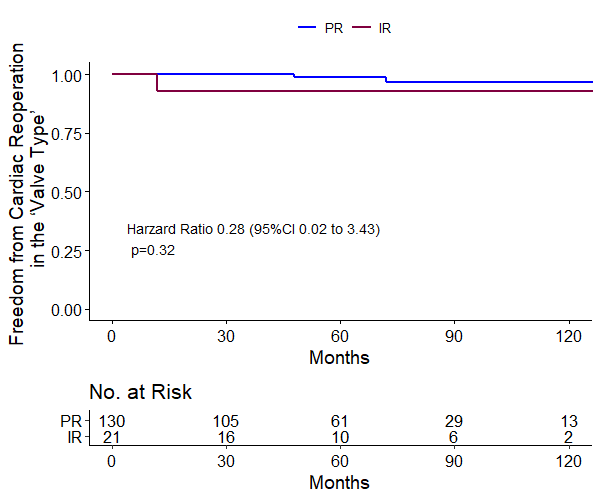
**S6-1 (35~40mm)**


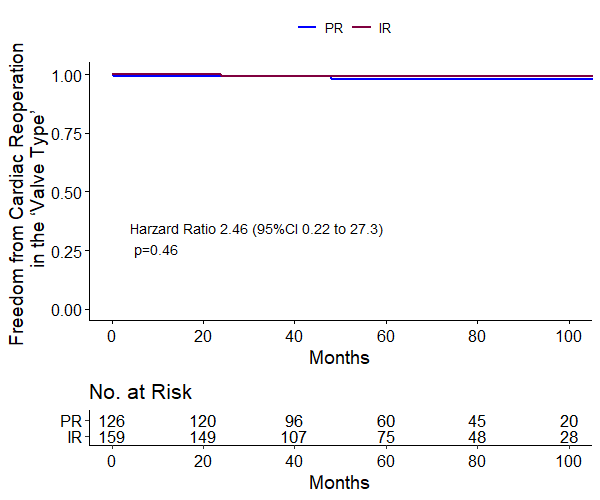
**S6-2 (>40mm)**

**Figure S9. Kaplan-Meier Freedom from All-cause Mortality in the ‘Aorta Type’, Stratified by Diameter and Procedures**

**S7-1 (45~52mm)**


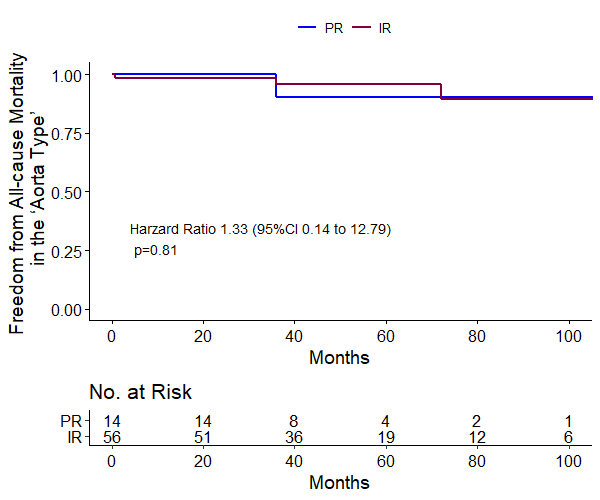


**S7-2 (52~60mm)**


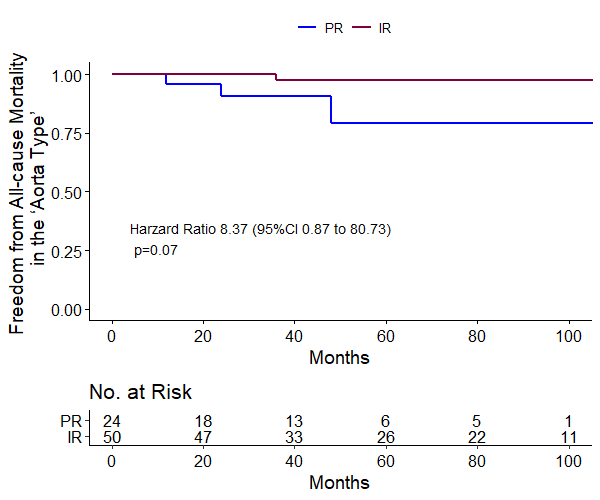


**Figure S10. Kaplan-Meier Freedom from Cardiac Reoperation in the ‘Aorta Type’, Stratified by Diameter and Procedures**

**S8-1 (45~52mm)**


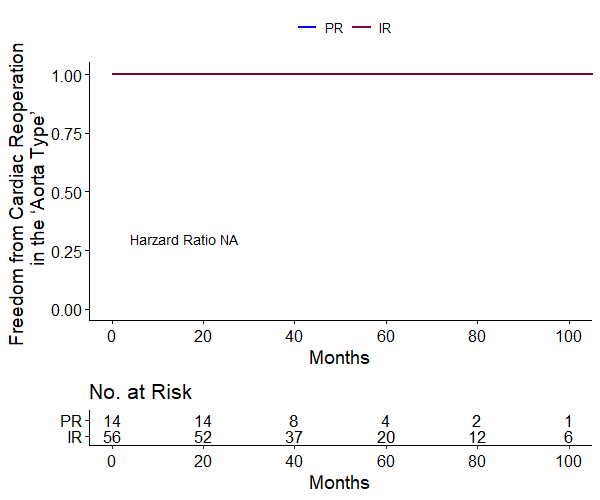


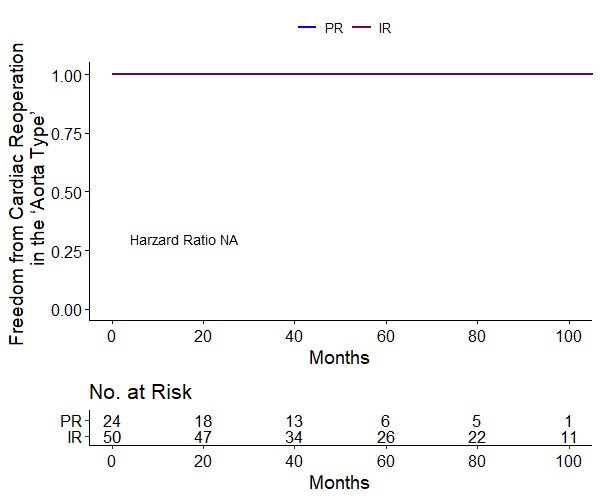
**S8-2 (52~60mm)**

**Figure S11. Kaplan-Meier Freedom from Cumulative Incidence of Stroke, Bleeding, NYHA function class II-IV, Significant aortic valve dysfunction in the ‘Valve Type’, Stratified by Diameter and Procedures**


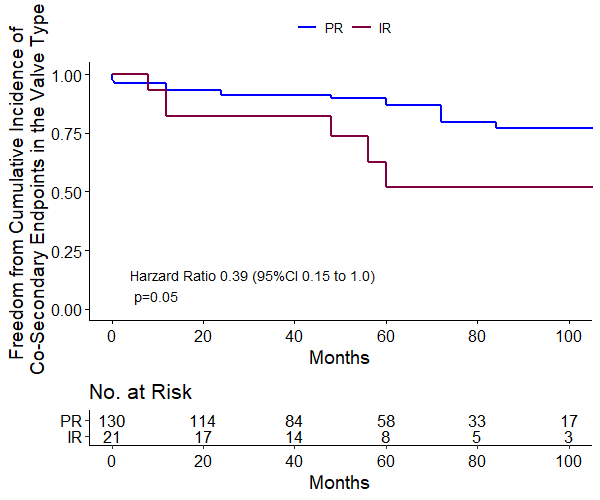
**S9-1 (35~40mm)**

**S9-2 (>40mm)**


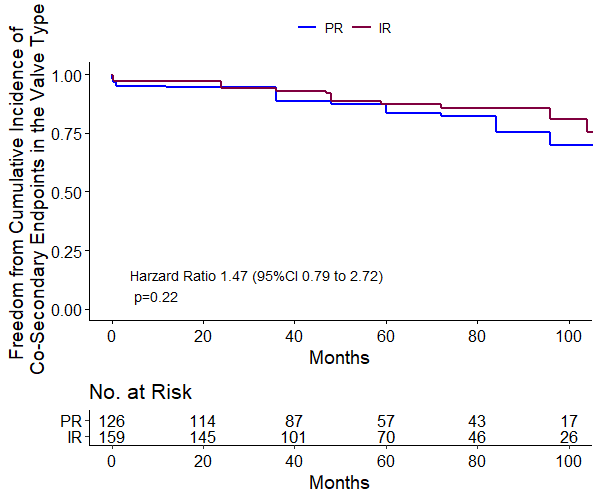


**Figure S12. Kaplan-Meier Freedom from NYHA function class II-IV in the ‘Valve Type’, Stratified by Diameter and Procedures**

**S10-1 (35~40mm)**


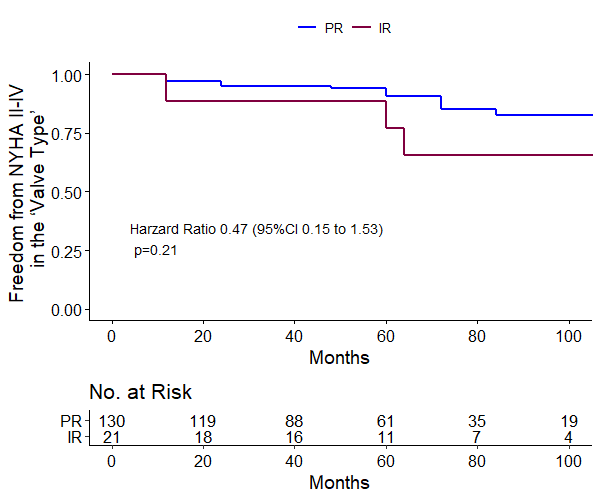


**S10-2 (>40mm)**


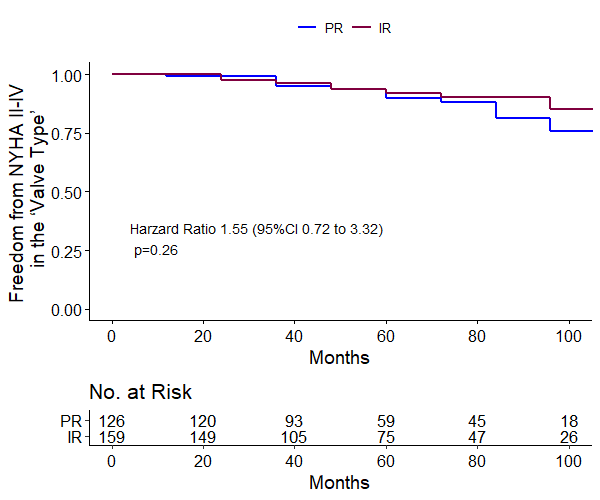


**Figure S13. Kaplan-Meier Freedom from Cumulative Incidence of Stroke, Bleeding, NYHA function class II-IV, Significant aortic valve dysfunction in the ‘Aorta Type’, Stratified by Diameter and Procedures**

**S11-1 (45~52mm)**


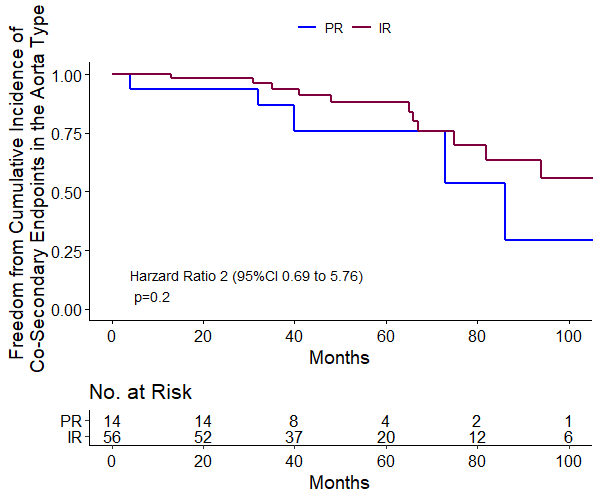


**S11-2 (52~60mm)**


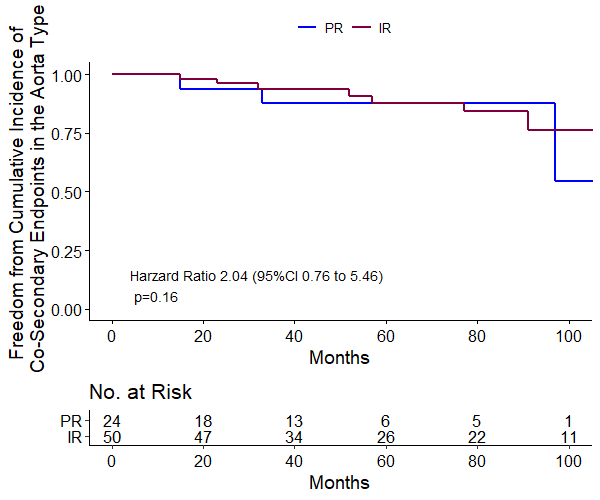


**Figure S14. Kaplan-Meier Freedom from NYHA function class II-IV in the ‘Aorta Type’, Stratified by Diameter and Procedures**

**S12-1 (45~52mm)**


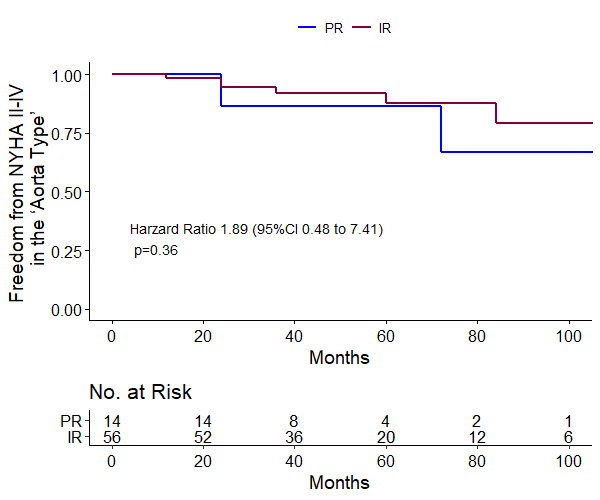


**S12-2 (52~60mm)**


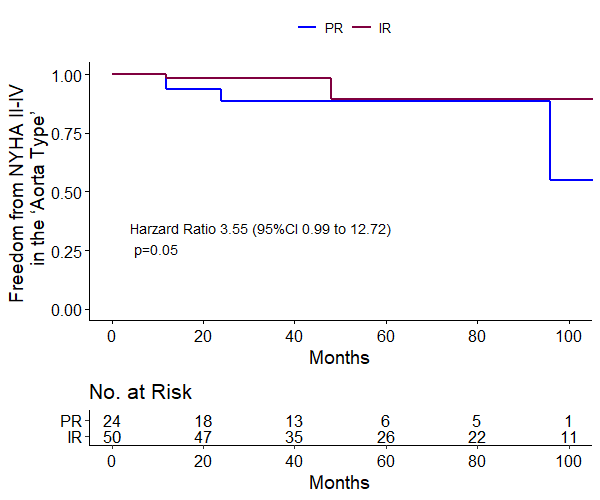


**Figure S15. Kaplan-Meier Freedom from Safety Endpoints in the ‘Valve Type’, Stratified by Diameter and Procedures**

**S13-1 (35~40mm)**


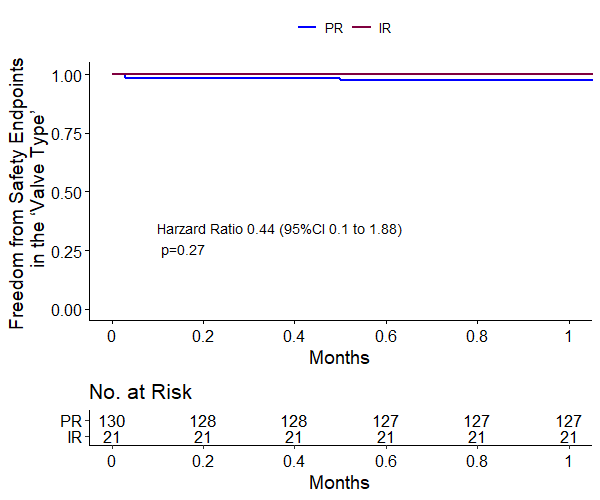


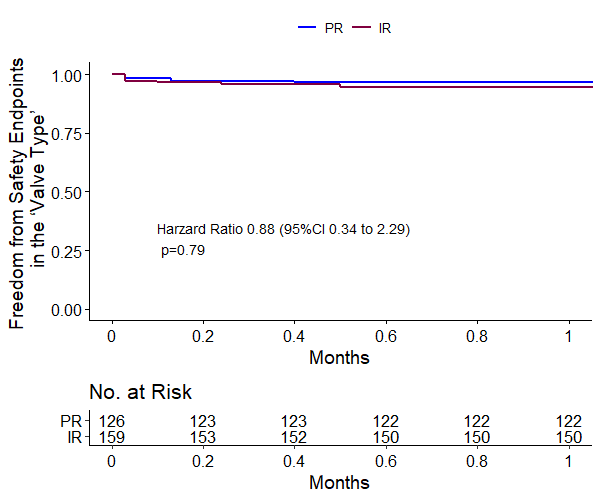
**S13-2 (>40mm)**

**Figure S16. Kaplan-Meier Freedom from Safety Endpoints in the ‘Aorta Type’, Stratified by Diameter and Procedures**

**S14-1 (45~52mm)**


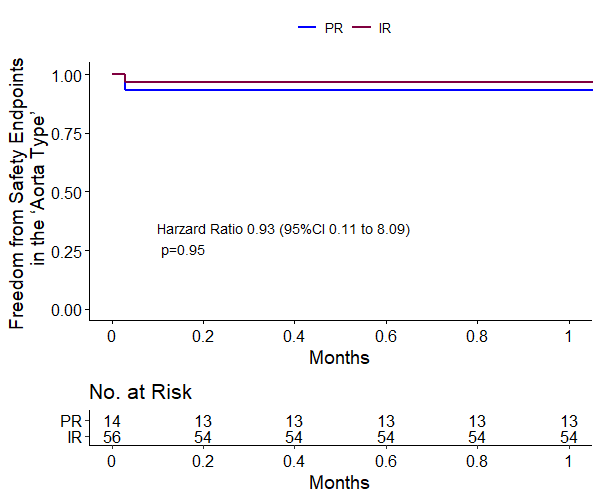


**S14-2 (52~60mm)**


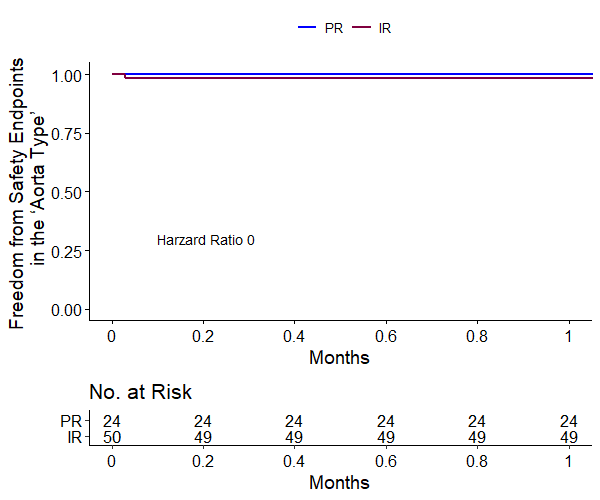


**Table S1.** **Models for Estimating Propensity Scores for ‘Valve Type’**

| **Variable** | **Overall Study Population** | **35-40 mm** | **>40 mm** |
| --- | --- | --- | --- |
| Intercept | 156.41 | -275.09 | 352.11 |
| Age | 0.02 | 0.11 | 0.02 |
| Year of Surgery | -0.06 | 0.16 | -0.16 |
| Male sex | -0.28 | 0.87 | -0.50 |
| Severe AS | -0.87 | -20.47 | -0.92 |
| Severe AR | -1.73 | -18.31 | -1.70 |
| Severe AS+AR | *NA* | *NA* | *NA* |
| Vmax | -0.02 | -0.02 | -0.01 |
| Max gradient | 0.00 | -0.02 | -0.03 |
| Mean gradient | 0.06 | 0.15 | 0.07 |
| Valve diameter | -0.09 | 0.05 | -0.11 |
| Sinuses diameter | -0.04 | -0.11 | -0.06 |
| Ascending diameter | 0.14 | 0.10 | -0.07 |
| Max (Sinuses, Asc) | -0.53 | -0.61 | -0.43 |
| BAV type | -0.73 | -1.42 | -0.77 |
| Hypertension | -0.61 | -2.10 | -0.83 |
| Diabetes mellitus | 0.33 | 17.58 | 0.39 |
| Coronary artery disease | -0.61 | -2.36 | -0.73 |
| Peripheral vascular disease | 0.24 | -17.03 | 1.43 |
| Cerebrovascular disease | 1.05 | 33.45 | 0.85 |
| Congestive heart failure | -0.07 | -3.47 | 0.07 |
| Atrial fibrillation | 2.48 | 18.86 | 2.58 |
| COPD | -1.59 | -37.19 | -2.69 |
| SBE | 0.09 | 18.77 | -1.43 |
| Chronic kidney disease | -2.00 | 17.48 | -15.59 |
| Renal dialysis | *NA* | *NA* | *NA* |
| Liver disease | -0.32 | 18.61 | -0.25 |
| Cancer | 13.10 | 1.33 | *NA* |
| History of smoking | 0.03 | -1.33 | 0.41 |
| Dissection | -0.48 | *NA* | -0.42 |
| Obesity | 1.40 | 2.18 | 1.16 |

△P = pressure gradient between the LV and aorta; AR = aortic regurgitation; AS = aortic stenosis; BAV = bicuspid aortic valve; CABG = coronary artery bypass graft; COPD = chronic obstructive pulmonary disease; IR = integrated aortic-valve-and-ascending-aortic replacement; PR = partial replacement; SBE = subacute bacterial endocarditis; V_max_ = maximum velocity

**Table S2.** **Models for Estimating Propensity Scores for ‘Aorta Type’**

| **Variable** | **Overall Study Population** | **45~50mm** | **>50mm** |
| --- | --- | --- | --- |
| Intercept | -614.36 | -995.29 | -544.89 |
| Age | -0.02 | 0.03 | -0.03 |
| Year of Surgery | 0.31 | 0.50 | 0.27 |
| Male sex | -1.02 | -1.31 | -0.97 |
| Moderate AS | -2.77 | -22.42 | -1.83 |
| Moderate AR | -17.74 | -21.12 | -17.96 |
| Moderate AS+AR | -18.10 | -20.49 | -18.30 |
| Valve diameter | -0.17 | -0.40 | -0.15 |
| Sinuses diameter | -0.07 | -0.16 | -0.04 |
| Ascending diameter | 0.03 |  |  |

AR = aortic regurgitation; AS = aortic stenosis

**Table S3.** **Baseline and Operative Characteristics of the ‘Valve Type’ Before and After Inverse Probability Weighting**

|  | **Valve Type (Unweighted)** | | | **Valve Type (Weighted)** | | |
| --- | --- | --- | --- | --- | --- | --- |
| **Characteristic** | **PR (N=329)** | **IR (N=173)** | **SMD** | **PR (N=333.3)** | **IR (N=178.7)** | **SMD** |
| Age (years) | 48.7±14.4 | 50.0±12.1 | -0.1 | 48.6±14.4 | 49.9±12.0 | -0.095 |
| Year of surgery (years) | 2015±3.1 | 2015.2±3.1 | -0.06 | 2015±3.1 | 2015.1±3.1 | -0.032 |
| Study period |  |  | 0.041 |  |  | 0.036 |
| 2002-2007 | 3(0.9%) | 1(0.6%) |  | 3.2(1.0%) | 1.1(0.6%) |  |
| 2008-2013 | 94(28.6%) | 48(27.7%) |  | 94.1(28.2%) | 50.4(28.2%) |  |
| 2014-2020 | 232(70.5%) | 124(71.7%) |  | 236(70.8%) | 127.2(71.2%) |  |
| Sex |  |  | 0.296 |  |  | 0.245 |
| male | 228(69.3%) | 144(83.2%) |  | 232.6(69.8%) | 145.5(81.4%) |  |
| female | 101(30.7%) | 29(16.8%) |  | 100.7(30.2%) | 33.2(18.6%) |  |
| Valvular disease |  |  | 0.204 |  |  | 0.173 |
| Severe AS | 166(50.5%) | 72(41.6%) |  | 166.6(50.0%) | 76.9(43%) |  |
| Severe AR | 151(45.9%) | 89(51.4%) |  | 155.7(46.7%) | 90.8(50.8%) |  |
| Severe AS+AR | 12(3.6%) | 12(7.0%) |  | 11(3.3%) | 11(6.2%) |  |
| AS/AR mild | 0 | 0 |  | 0 | 0 |  |
| Moderate AS | 0 | 0 |  | 0 | 0 |  |
| Moderate AR | 0 | 0 |  | 0 | 0 |  |
| Moderate AS+AR | 0 | 0 |  | 0 | 0 |  |
| AS |  |  |  |  |  |  |
| Aortic Vmax | 504.0±76.1 | 500.4±70.9 | 0.048 | 504.2±75.5 | 500±71.1 | 0.057 |
| Aortic mean gradient | 63.3±21.2 | 60.5±19.0 | 0.137 | 63.2±21.0 | 60.9±19.1 | 0.113 |
| AR |  |  |  |  |  |  |
| EROA | 45.2±15.7 | 52.4±19.6 | -0.42 | 45.1±15.6 | 52.6±19.5 | -0.439 |
| Vena Contracta Width | 7.6±1.7 | 8.0±1.2 | -0.26 | 7.6±1.7 | 8.1±1.2 | -0.323 |
| AS+AR |  |  |  |  |  |  |
| Aortic Vmax | 504±75.7 | 455.3±41.5 | 0.737 | 505.6±72.8 | 456.4±40.2 | 0.775 |
| Aortic mean gradient | 70.1±31.4 | 49.2±7.2 | 0.81 | 70.5±30.1 | 49.2±7.0 | 0.863 |
| EROA | 43.0±10.8 | 41±13.5 | 0.169 | 43.1±10.4 | 40.8±12.6 | 0.205 |
| Vena Contracta Width | 7.4±0.9 | 7.4±1.1 | 0 | 7.4±0.9 | 7.3±1.1 | 0.103 |
| Valve diameter | 23.3±3.1 | 25.6±4.0 | -0.668 | 23.4±3.1 | 25.4±4.0 | -0.581 |
| Sinuses diameter | 33.1±5.4 | 40.3±8.6 | -1.077 | 33.3±5.5 | 39.7±8.5 | -0.954 |
| Ascending aorta diameter | 38.5±5.8 | 49.6±7.7 | -1.701 | 38.7±5.9 | 48.9±7.5 | -1.566 |
| Max (Sinus, Asc) | 39.2±5.4 | 50.9±7.2 | -1.922 | 39.4±5.5 | 50.2±7.0 | -1.778 |
| BAV type |  |  | 0.159 |  |  | 0.115 |
| type 0 | 162(49.2%) | 71(41.0%) |  | 160.9(48.3%) | 75.7(42.4%) |  |
| type 1 | 160(48.6%) | 97(56.1%) |  | 165.2(49.6%) | 98.1(54.9%) |  |
| type 2 | 7(2.1%) | 5(2.9%) |  | 7.2(2.1%) | 4.9(2.7%) |  |
| Coexisting condition (%) |  |  |  |  |  |  |
| Hypertension | 81(24.6%) | 57(32.9%) | 0.169 | 82.6(24.8%) | 56.9(31.8%) | 0.142 |
| Diabetes mellitus | 23(7.0%) | 14(8.1%) | 0.024 | 22.3(6.7%) | 13.4(7.5%) | 0.014 |
| Coronary artery disease | 34(10.3%) | 15(8.7%) | 0.039 | 34.6(10.4%) | 15.2(8.5%) | 0.047 |
| Peripheral vascular disease | 7(2.1%) | 3(1.7%) | 0 | 6.8(2.0%) | 3(1.7%) | 0 |
| Cerebrovascular disease | 10(3.0%) | 5(2.9%) | 0 | 10(3.0%) | 5.3(3%) | 0 |
| Congestive heart failure | 159(48.3%) | 78(45.1%) | 0.053 | 164.3(49.3%) | 83.2(46.6%) | 0.044 |
| Atrial fibrillation | 9(2.7%) | 3(1.7%) | 0.035 | 8.5(2.6%) | 3.2(1.8%) | 0.021 |
| COPD | 2(0.6%) | 1(0.6%) | 0 | 1.9(0.6%) | 0.9(0.5%) | 0 |
| SBE | 23(7.0%) | 2(1.2%) | 0.237 | 22.2(6.7%) | 1.8(1%) | 0.237 |
| Chronic kidney disease | 1(0.3%) | 2(1.2%) | 0.051 | 0.9(0.3%) | 1.8(1%) | 0.041 |
| Renal dialysis | 0 | 0 |  | 0 | 0 |  |
| Liver disease | 8(2.4%) | 10(5.8%) | 0.149 | 7.3(2.2%) | 8.9(5%) | 0.129 |
| Cancer | 2(0.6%) | 0 | 0.025 | 1.9(0.6%) | 0 | 0.022 |
| History of smoking | 98(29.8%) | 58(33.5%) | 0.068 | 99.9(30%) | 60.2(33.7%) | 0.068 |
| Dissection | 1(0.3%) | 3(1.7%) | 0.106 | 1.2(0.4%) | 3.1(1.7%) | 0.099 |
| Obesity | 15(4.6%) | 11(6.4%) | 0.058 | 14.1(4.2%) | 9.5(5.3%) | 0.03 |
| Concomitant procedure |  |  |  |  |  |  |
| CABG | 18(5.5%) | 13(7.5%) | 0.063 | 18.8(5.6%) | 13.7(7.7%) | 0.062 |
| TVP | 0 | 0 |  | 0 | 0 |  |
| AFA | 3(0.9%) | 1(0.6%) | 0 | 2.7(0.8%) | 1(0.6%) | 0 |
| Bentall | 0 | 129(74.6%) |  | 0 | 131.7(73.7%) |  |
| Prosthetic type |  |  | 0.34 |  |  | 0.34 |
| Mechanical | 271(82.4%) | 164(94.8%) |  | 273.6(82.1%) | 169.1(94.6%) |  |
| Biological | 58(17.6%) | 9(5.2%) |  | 59.7(17.9%) | 9.6(5.4%) |  |

△P, pressure gradient between the LV and aorta; AFA = atrial fibrillation ablation; AR = aortic regurgitation; AS = aortic stenosis; BAV = bicuspid aortic valve; CABG = coronary artery bypass graft; COPD = chronic obstructive pulmonary disease; ERO = effective regurgitant orifice; IR = integrated aortic-valve-and-ascending-aortic replacement; PR = partial replacement; SBE = subacute bacterial endocarditis; TVP = tricuspid valve plasty; Vmax = maximum velocity

**Table S4.** **Baseline and Operative Characteristics of the ‘Aorta Type’ Before and After Inverse Probability Weighting**

|  | **Aorta Type (Unweighted)** | | | **Aorta Type (Weighted)** | | |
| --- | --- | --- | --- | --- | --- | --- |
| **Characteristic** | **PR (N=41)** | **IR (N=128)** | **SMD** | **PR (N=47.4)** | **IR (N=132.4)** | **SMD** |
| Age (years) | 52.3±9.0 | 50.4±12.1 | 0.17 | 52.0±8.9 | 50.5±11.8 | 0.13 |
| Year of surgery (years) | 2016.4±2.5 | 2015.2±2.8 | 0.44 | 2015.8±2.7 | 2015.3±2.7 | 0.18 |
| Study period |  |  | 0.242 |  |  | 0.03 |
| 2002-2007 | 0 | 0 |  | 0 | 0 |  |
| 2008-2013 | 7(17.1%) | 40(31.2%) |  | 12.4(26.1%) | 38.3(28.9%) |  |
| 2014-2020 | 34(82.9%) | 88(68.8%) |  | 35(73.9%) | 94.1(71.1%) |  |
| Sex |  |  | 0.425 |  |  | 0.27 |
| male | 25(61.0%) | 106(82.8%) |  | 31.2(65.8%) | 105.9(80%) |  |
| female | 16(39.0%) | 22(17.2%) |  | 16.2(34.2%) | 26.5(20%) |  |
| Valvular disease |  |  | 0.76 |  |  | 0.63 |
| Severe AS | 0 | 0 |  | 0 | 0 |  |
| Severe AR | 0 | 0 |  | 0 | 0 |  |
| Severe AS+AR | 0 | 0 |  | 0 | 0 |  |
| AS/AR mild | 38(92.7%) | 68(53.1%) |  | 42.6(89.9%) | 78.1(59%) |  |
| Moderate AS | 3(7.3%) | 34(26.6%) |  | 4.8(10.1%) | 31.5(23.8%) |  |
| Moderate AR | 0 | 18(14.1%) |  | 0 | 15.8(11.9%) |  |
| Moderate AS+AR | 0 | 8(6.3%) |  | 0 | 7(5.3%) |  |
| Valve diameter | 22.7±2.0 | 24.0±2.4 | -0.56 | 22.9±2.0 | 23.8±2.4 | -0.39 |
| Sinuses diameter | 36.0±4.4 | 38.6±8.4 | -0.34 | 36.3±4.4 | 38.3±8.1 | -0.27 |
| Ascending aorta diameter | 53.7±4.5 | 52.7±8.6 | 0.127 | 53.7±4.4 | 52.7±8.4 | 0.13 |
| Max (Sinus, Asc) | 53.7±4.5 | 53.6±7.5 | 0.014 | 53.7±4.4 | 53.5±7.3 | 0.03 |
| BAV type |  |  | 0.014 |  |  | 0.01 |
| type 0 | 17(41.5%) | 50(39.1%) |  | 27.6(58.2%) | 79.7 |  |
| type 1 | 24(58.5%) | 78(60.9%) |  | 19.8(41.8%) | 52.6 |  |
| type 2 | 0 | 0 |  | 0 |  |  |
| Coexisting condition (%) |  |  |  |  |  |  |
| Hypertension | 15(36.6%) | 47(36.7%) | 0 | 18.1(38.2) | 49.3(37.2%) | 0 |
| Diabetes mellitus | 0 | 6(4.7%) | 0.143 | 0 | 5.9(4.5%) | 0.15 |
| Coronary artery disease | 8(19.5%) | 17(13.3%) | 0.112 | 9.2(19.4%) | 17.9(13.5%) | 0.11 |
| Peripheral vascular disease | 1(2.4%) | 2(1.6%) | 0 | 1.2(2.5%) | 1.9(1.4%) | 0 |
| Cerebrovascular disease | 2(4.9%) | 6(4.7%) | 0 | 3.6(7.6%) | 5.2(3.9%) | 0.09 |
| Congestive heart failure | 12(29.3%) | 46(35.9%) | 0.091 | 11.4(24.1%) | 48.7(36.8%) | 0.21 |
| Atrial fibrillation | 1(2.4%) | 4(3.1%) | 0 | 0.8(1.7%) | 4.1(3.1%) | 0 |
| COPD | 0 | 0 |  | 0 | 0 | 0 |
| SBE | 0 | 0 |  | 0 | 0 | 0 |
| Chronic kidney disease | 0 | 3(2.3%) | 0.048 | 0 | 2.9(2.2%) | 0.05 |
| Renal dialysis | 0 | 1(0.8%) | 0 | 0 | 1(0.8%) | 0 |
| Liver disease | 3(7.3%) | 3(2.3%) | 0.156 | 3.2(6.8%) | 3.9(2.9%) | 0.11 |
| Cancer | 2(4.9%) | 0 | 0.261 | 2.6(5.5%) | 0 | 0.31 |
| History of smoking | 13(31.7%) | 51(39.8%) | 0.116 | 16.5(34.8%) | 53.8(40.6%) | 0.08 |
| Dissection | 0 | 7(5.5%) | 0.167 | 0 | 6.5(4.9%) | 0.16 |
| Obesity | 2(4.9%) | 13(10.2%) | 0.111 | 2.6(5.5%) | 12.8(9.7%) | 0.09 |
| Concomitant procedure |  |  |  |  |  |  |
| CABG | 2(4.9%) | 14(10.9%) | 0.131 | 3.6(7.6%) | 14(10.6%) | 0.05 |
| TVP | 0 | 0 |  | 0 | 0 |  |
| AFA | 0 | 0 |  | 0 | 0 |  |
| Bentall | 0 | 91(71.1%) |  | 0 | 96.5(72.9%) |  |
| Prosthetic type |  |  |  |  |  |  |
| Mechanical |  | 122(95.3%) |  |  | 126.4(95.5%) |  |
| Biological |  | 6(4.7%) |  |  | 6(4.5%) |  |

△P, pressure gradient between the LV and aorta; AFA = atrial fibrillation ablation; AR = aortic regurgitation; AS = aortic stenosis; BAV = bicuspid aortic valve; CABG = coronary artery bypass graft; COPD = chronic obstructive pulmonary disease; ERO = effective regurgitant orifice; IR = integrated aortic-valve-and-ascending-aortic replacement; PR = partial replacement; SBE = subacute bacterial endocarditis; SMD = standard mean difference; TVP = tricuspid valve plasty; Vmax = maximum velocity

**Table S5. Aortic Diameter Distribution and Aortic Valve Function between the ‘Valve Type’ and the ‘Aorta Type’**

|  | Valve Type | Aorta Type | p |
| --- | --- | --- | --- |
|  | (N = 502) | (N = 169) |  |
| Aortic diameter | 43.2±8.2 | 53.6±6.9 | <0.0001 |
| Sinuses Diameter | 35.6±7.5 | 38.0±7.7 | 0.0003 |
| Ascending aortic diameter | 42.3±8.4 | 52.9±7.8 | <0.0001 |
| Ratio (Ascending/Sinuses) | 1.2±0.3 | 1.4±0.3 | <0.0001 |
| Significant dysfunction |  |  |  |
| Severe aortic stenosis | 238(47.4%) |  |  |
| Severe aortic regurgitation | 240(47.8%) |  |  |
| Severe aortic stenosis and regurgitation | 24(4.8%) |  |  |
| Insignificant dysfunction |  |  |  |
| Moderate aortic stenosis |  | 31(18.3%) |  |
| Moderate aortic regurgitation |  | 20(11.8%) |  |
| Moderate aortic stenosis and regurgitation | | 9(5.3%) |  |
| None or trace dysfunction |  | 109(64.5%) |  |
| Sievers' classification |  |  | 0.03 |
| Type 0 | 233(46.4%) | 67(39.6%) |  |
| Type 1 | 257(51.2%) | 102(60.4%) |  |
| Type 2 | 12(2.4%) | 0(0.0%) |  |

**Table S6. Diameter-Group (35-40 and >40) Differences in Primary Endpoints in ‘Valve Type’** *

| **Variable** | **35 - 40 mm** | | | **> 40 mm** | | |
| --- | --- | --- | --- | --- | --- | --- |
|  | **PR (N=130.1)** | **IR (N=20.7)** | **p value** | **PR (N=126.5)** | **IR (N=158.9)** | **p value** |
| Hazard ratio (95% CI) |  |  |  |  |  |  |
| Weighted PH model | 1.19 (0.14 to 9.80) | Reference | 0.87 | 3.22 (1.52 to 6.85) | Reference | 0.002 |
| Weighted PH model, with multivariable adjustment^†^ | 0.91 (0.14 to 5.86) | Reference | 0.92 | 3.22 (1.51 to 6.84) | Reference | 0.002 |
| Weighted PH model, with surgeon as random effect | 1.19 (0.21 to 6.77) | Reference | 0.85 | 3.21 (1.49 to 6.89) | Reference | 0.003 |
| **5 years** | | | | | | |
| Incidence (%) | 5.48% | 7.04% | 0.78 | 11.98% | 4.65% | 0.02 |
| RMST 5 years (95% CI) |  |  |  |  |  |  |
| Difference (months) | 0.95 (-4.96 to 6.85) | Reference | 0.75 | -2.15 (-5.18 to 0.88) | Reference | 0.17 |
| Ratio | 1.02 (0.92 to 1.13) | Reference | 0.76 | 0.96 (0.91 to 1.02) | Reference | 0.17 |
| Ratio of RMSL | 0.68 (0.09 to 5.31) | Reference | 0.72 | 1.96 (0.76 to 5.04) | Reference | 0.16 |
| **10 years** | | | | | | |
| Incidence (%) | 7.7% | 7.04% | 0.9 | 17.49% | 5.28% | 0.001 |
| RMST (95% CI) ‡ |  |  |  |  |  |  |
| Difference — months | -2.3 (-16.2 to 11.6) | Reference | 0.74 | -11.3 (-19.6 to -3.0) | Reference | 0.007 |
| Ratio | 0.98 (0.87 to 1.11) | Reference | 0.74 | 0.89 (0.83 to 0.97) | Reference | 0.009 |
| Ratio of RMSL | 1.34(0.18 to 9.8) | Reference | 0.77 | 2.65(1.3 to 5.6) | Reference | 0.01 |

*The overall numbers of patients in each group are not necessarily integers owing to inverse probability weighting.
† The analysis was adjusted for sinuses diameter.
‡ The RMST is the average duration of survival in a cohort over a prespecified follow-up period (5 years and 10 years were reported here), as estimated by the area under the curve. The difference in the RMST is the average number of additional months gained in the treatment group (i.e., IR group minus PR group). The RMTL refers to the average number of days of life lost over a prespecified follow-up period; a ratio of more than 1.00 indicates that the treatment increased events incidence (or decreased the survival rate).

BAV = bicuspid aortic valve; IR = integrated aortic-valve-and-ascending-aortic replacement; RMST = restricted mean survival time; RMTL = restricted mean time lost; PH = proportional-hazards; PR = partial replacement

**Table S7. Diameter-Group (35-45 and >45) Differences in Primary Endpoints in ‘Valve Type’** *

| **Variable** | **35 - 45 mm** | | | **> 45 mm** | | |
| --- | --- | --- | --- | --- | --- | --- |
|  | **PR (N=212.6)** | **IR (N=52.2)** | **p value** | **PR (N=44)** | **IR (N=127.4)** | **p value** |
| Hazard ratio (95% CI) |  |  |  |  |  |  |
| Weighted PH model | 2.17 (0.5 to 9.41) | Reference | 0.3 | 3.61 (1.51 to 8.66) | Reference | 0.004 |
| Weighted PH model, with multivariable adjustment^†^ | 2.41 (0.58 to 9.98) | Reference | 0.22 | 3.32 (1.46 to 7.52) | Reference | 0.004 |
| Weighted PH model, with surgeon as random effect | 2.19 (0.57 to 8.45) | Reference | 0.25 | 3.60 (1.44 to 9.01) | Reference | 0.006 |
| **5 years** | | | | | | |
| Incidence (%) | 6.85% | 2.79% | 0.27 | 17.52% | 5.81% | 0.019 |
| RMST 5 years (95% CI) |  |  |  |  |  |  |
| Difference (months) | -1.72 (-4.4 to 0.97) | Reference | 0.21 | -2.26 (-7.4 to 2.88) | Reference | 0.39 |
| Ratio | 0.97 (0.93 to 1.02) | Reference | 0.21 | 0.96 (0.88 to 1.05) | Reference | 0.40 |
| Ratio of RMSL | 2.5 (0.34 to 18.65) | Reference | 0.37 | 1.84 (0.55 to 6.18) | Reference | 0.32 |
| **10 years** | | | | | | |
| Incidence (%) | 9.9% | 4.69% | 0.24 | 25.28% | 5.81% | 0.0003 |
| RMST (95% CI) ‡ |  |  |  |  |  |  |
| Difference — months | -6.11 (-14.71 to 2.48) | Reference | 0.16 | -14.53 (-27.99 to -1.07) | Reference | 0.03 |
| Ratio | 0.95 (0.88 to 1.02) | Reference | 0.16 | 0.87 (0.76 to 1.0) | Reference | 0.04 |
| Ratio of RMSL | 2.11 (0.53 to 8.34) | Reference | 0.29 | 2.96 (1.23 to 7.14) | Reference | 0.02 |

*The overall numbers of patients in each group are not necessarily integers owing to inverse probability weighting.
† The analysis was adjusted for sinuses diameter.
‡ The RMST is the average duration of survival in a cohort over a prespecified follow-up period (5 years and 10 years were reported here), as estimated by the area under the curve. The difference in the RMST is the average number of additional months gained in the treatment group (i.e., IR group minus PR group). The RMTL refers to the average number of days of life lost over a prespecified follow-up period; a ratio of more than 1.00 indicates that the treatment increased events incidence (or decreased the survival rate).

BAV = bicuspid aortic valve; IR = integrated aortic-valve-and-ascending-aortic replacement; RMST = restricted mean survival time; RMTL = restricted mean time lost; PH = proportional-hazards; PR = partial replacement

**Table S8. Diameter-Group (45-50 and >50) Differences in Primary Endpoints in ‘Aorta Type’** *

| **Variable** | **45 - 50 mm** | | | **> 50 mm** | | |
| --- | --- | --- | --- | --- | --- | --- |
|  | **PR (N=9.3)** | **IR (N=35.9)** | **p value** | **PR (N=37.1)** | **IR (N=84.4)** | **p value** |
| Hazard ratio (95% CI) |  |  |  |  |  |  |
| Weighted PH model | 4.25 (0.28 to 63.83) | Reference | 0.296 | 2.81 (0.66 to 12.03) | Reference | 0.16 |
| Weighted PH model, with multivariable adjustment^†^ | 6.67 (0.8 to 55.56) | Reference | 0.08 | 2.83 (0.66 to 12.22) | Reference | 0.16 |
| Weighted PH model, with surgeon as random effect | 2.81 (0.16 to 47.91) | Reference | 0.47 | 3.13 (0.68 to 14.45) | Reference | 0.14 |
| **5 years** | | | | | | |
| Incidence (%) | 11.64% | 2.84% | 0.27 | 10.18% | 2.3% | 0.069 |
| RMST 5 years (95% CI) |  |  |  |  |  |  |
| Difference (months) | -3.11 (-10.47 to 4.25) | Reference | 0.41 | -2.43 (-6.78 to 1.93) | Reference | 0.27 |
| Ratio | 0.95 (0.83 to 1.08) | Reference | 0.42 | 0.96 (0.89 to 1.04) | Reference | 0.28 |
| Ratio of RMSL | 4.5 (0.33 to 62.24) | Reference | 0.26 | 3.1 (0.51 to 18.99) | Reference | 0.22 |
| **10 years** | | | | | | |
| Incidence (%) | 11.64% | 2.84% | 0.27 | 10.18% | 4.67% | 0.27 |
| RMST 10 year (95% CI) ‡ |  |  |  |  |  |  |
| Difference — months | -6.48 (-21.81 to 8.85) | Reference | 0.41 | -6.97 (-19.89 to 5.96) | Reference | 0.29 |
| Ratio | 0.92 (0.76 to 1.12) | Reference | 0.42 | 0.94 (0.84 to 1.06) | Reference | 0.30 |
| Ratio of RMSL | 4.5 (0.33 to 62.24) | Reference | 0.26 | 2.63 (0.56 to 12.25) | Reference | 0.22 |

*The overall numbers of patients in each group are not necessarily integers owing to inverse probability weighting.
† The analysis was adjusted for sinuses diameter.
‡ The RMST is the average duration of survival in a cohort over a prespecified follow-up period (5 years and 10 years were reported here), as estimated by the area under the curve. The difference in the RMST is the average number of additional months gained in the treatment group (i.e., IR group minus PR group). The RMTL refers to the average number of days of life lost over a prespecified follow-up period; a ratio of more than 1.00 indicates that the treatment increased events incidence (or decreased the survival rate).

BAV = bicuspid aortic valve; IR = integrated aortic-valve-and-ascending-aortic replacement; RMST = restricted mean survival time; RMTL = restricted mean time lost; PH = proportional-hazards; PR = partial replacement

**Table S9. Diameter-Group (45-55 and >55) Differences in Primary Endpoints in ‘Aorta Type’** *

| **Variable** | **45 - 55 mm** | | | **> 55 mm** | | |
| --- | --- | --- | --- | --- | --- | --- |
|  | **PR (N=32.6)** | **IR (N=80.6)** | **p value** | **PR (N=13.8)** | **IR (N=39.8)** | **p value** |
| Hazard ratio (95% CI) |  |  |  |  |  |  |
| Weighted PH model | 2.17 (0.48 to 9.7) | Reference | 0.31 | 6.85 (0.52 to 90.68) | Reference | 0.14 |
| Weighted PH model, with multivariable adjustment^†^ | 2.23 (0.51 to 9.72) | Reference | 0.29 | 8.0 (0.5 to 127.5) | Reference | 0.14 |
| Weighted PH model, with surgeon as random effect | 2.17 (0.47 to 10.06) | Reference | 0.32 | 5.98 (0.41 to 86.67) | Reference | 0.19 |
| **5 years** | | | | | | |
| Incidence (%) | 9.63% | 3.71% | 0.23 | 12.44% | 0 | 0.03 |
| RMST 5 years (95% CI) |  |  |  |  |  |  |
| Difference (months) | -2.95 (-8.22 to 2.31) | Reference | 0.27 | -2.0 (-5.58 to 1.58) | Reference | 0.27 |
| Ratio | 0.95 (0.86 to 1.04) | Reference | 0.28 | 0.97 (0.91 to 1.03) | Reference | 0.28 |
| Ratio of RMSL | 2.81 (0.58 to 13.59) | Reference | 0.20 | Inf | Reference | NA |
| **10 years** | | | | | | |
| Incidence (%) | 9.63% | 4.97% | 0.38 | 12.44% | 2.41% | 0.15 |
| RMST (95% CI) ‡ |  |  |  |  |  |  |
| Difference — months | -5.77 (-19.87 to 8.33) | Reference | 0.42 | -12.0 (-33.47 to 9.47) | Reference | 0.27 |
| Ratio | 0.95 (0.83 to 1.08) | Reference | 0.43 | 0.9 (0.74 to 1.10) | Reference | 0.30 |
| Ratio of RMSL | 1.92 (0.46 to 7.98) | Reference | 0.37 | Inf | Reference | NA |

*The overall numbers of patients in each group are not necessarily integers owing to inverse probability weighting.
† The analysis was adjusted for sinuses diameter.
‡ The RMST is the average duration of survival in a cohort over a prespecified follow-up period (5 years and 10 years were reported here), as estimated by the area under the curve. The difference in the RMST is the average number of additional months gained in the treatment group (i.e., IR group minus PR group). The RMTL refers to the average number of days of life lost over a prespecified follow-up period; a ratio of more than 1.00 indicates that the treatment increased events incidence (or decreased the survival rate).

BAV = bicuspid aortic valve; IR = integrated aortic-valve-and-ascending-aortic replacement; RMST = restricted mean survival time; RMTL = restricted mean time lost; PH = proportional-hazards; PR = partial replacement

**Table S10. 5-year Subdistribution Hazard of Mortality and Reoperation among PR versus IR in the ‘Valve Type’**

| **Mortality** | | | | |
| --- | --- | --- | --- | --- |
| **Diameter Group** | **HR** | **95%CI** | **PR** | **IR** |
| 35-40 mm | **Inf** | **Inf** | **4.58%** | **0** |
| > 40 mm | **2.74** | **1.24 to 6.07** | **9.32%** | **4.58%** |
| **Reoperation** | | | | |
| **Diameter Group** | **HR** | **95%CI** | **PR** | **IR** |
| 35-40 mm | **0.28** | **0.02 to 3.43** | **0.76%** | **6.25%** |
| > 40 mm | **2.46** | **0.22 to 27.3** | **1.69%** | **0.65%** |

CI = confidence interval; HR = hazard ratio

**Table S11. 10-year Subdistribution Hazard of Mortality and Reoperation among PR versus IR in the ‘Valve Type’**

| **Mortality** | | | | |
| --- | --- | --- | --- | --- |
| **Diameter Group** | **HR** | **95%CI** | **PR** | **IR** |
| 35-40 mm | **Inf** | **Inf** | **6.11%** | **0** |
| > 40 mm | **2.74** | **1.24 to 6.07** | **15.25%** | **5.23%** |
| **Reoperation** | | | | |
| **Diameter Group** | **HR** | **95%CI** | **PR** | **IR** |
| 35-40 mm | **0.28** | **0.02 to 3.43** | **1.53%** | **6.25%** |
| > 40 mm | **2.46** | **0.22 to 27.3** | **1.69%** | **0.65%** |

CI = confidence interval; HR = hazard ratio

**Table S12. 5-year Subdistribution Hazard of Mortality and Reoperation among PR versus IR in the ‘Aorta Type’**

| **Mortality** | | | | |
| --- | --- | --- | --- | --- |
| **Diameter Group** | **HR** | **95%CI** | **PR** | **IR** |
| 45-52 mm | **1.33** | **0.14 to 12.79** | **7.14%** | **3.51%** |
| 52-60 mm | **8.37** | **0.87 to 80.73** | **13.64%** | **2.04%** |
| **Reoperation** | | | | |
| **Diameter Group** | **HR** | **95%CI** | **PR** | **IR** |
| 45-52 mm | **NA** | **NA** | **0** | **0** |
| 52-60 mm | **NA** | **NA** | **0** | **0** |

CI = confidence interval; HR = hazard ratio

**Table S13. 10-year Subdistribution Hazard of Mortality and Reoperation among PR versus IR in the ‘Aorta Type’**

| **Mortality** | | | | |
| --- | --- | --- | --- | --- |
| **Diameter Group** | **HR** | **95%CI** | **PR** | **IR** |
| 45-52 mm | **1.33** | **0.14 to 12.79** | **7.14%** | **5.26%** |
| 52-60 mm | **8.37** | **0.87 to 80.73** | **13.64%** | **2.04%** |
| **Reoperation** | | | | |
| **Diameter Group** | **HR** | **95%CI** | **PR** | **IR** |
| 45-52 mm | **NA** | **NA** | **0** | **0** |
| 52-60 mm | **NA** | **NA** | **0** | **0** |

CI = confidence interval; HR = hazard ratio

**Table S14. 5-Year Subdistribution Hazard of Stroke, Bleeding, NYHA Function Class II-IV or Significant Prosthetic Valve Dysfunction among PR versus IR in the ‘Valve Type’**

| **Stroke** | | | | |
| --- | --- | --- | --- | --- |
| **Diameter Group** | **HR** | **95%CI** | **PR** | **IR** |
| 35-40 mm | **0** | **0** | **0** | **6.25%** |
| > 40 mm | **0.59** | **0.06 to 6.4** | **0.85%** | **1.31%** |
| **Bleeding** | | | | |
| **Diameter Group** | **HR** | **95%CI** | **PR** | **IR** |
| 35-40 mm | **0.52** | **0.07 to 3.97** | **3.05%** | **6.25%** |
| > 40 mm | **1.27** | **0.31 to 5.18** | **3.39%** | **2.61%** |
| **NYHA function class II-IV** | | | | |
| **Diameter Group** | **HR** | **95%CI** | **PR** | **IR** |
| 35-40 mm | **0.50** | **0.16 to 1.57** | **6.87%** | **18.75%** |
| > 40 mm | **1.49** | **0.72 to 3.1** | **6.78%** | **5.23%** |
| **Significant Prosthetic Valve Dysfunction** | | | | |
| **Diameter Group** | **HR** | **95%CI** | **PR** | **IR** |
| 35-40 mm | **Inf** | **Inf** | **0.76%** | **0** |
| > 40 mm | **0.61** | **0.11 to 3.46** | **1.69%** | **1.96%** |

CI = confidence interval; HR = hazard ratio

**Table S15. 10-Year Subdistribution Hazard of Stroke, Bleeding, NYHA function class II-IV, or Significant Prosthetic Valve Dysfunction among PR versus IR in the ‘Valve Type’**

| **Stroke** | | | | |
| --- | --- | --- | --- | --- |
| **Diameter Group** | **HR** | **95%CI** | **PR** | **IR** |
| 35-40 mm | **0** | **0** | **0** | **6.25%** |
| > 40 mm | **0.59** | **0.06 to 6.4** | **0.85%** | **1.31%** |
| **Bleeding** | | | | |
| **Diameter Group** | **HR** | **95%CI** | **PR** | **IR** |
| 35-40 mm | **0.52** | **0.07 to 3.97** | **3.05%** | **6.25%** |
| > 40 mm | **1.27** | **0.31 to 5.18** | **3.39%** | **2.61%** |
| **NYHA function class II-IV** | | | | |
| **Diameter Group** | **HR** | **95%CI** | **PR** | **IR** |
| 35-40 mm | **0.50** | **0.16 to 1.57** | **9.92%** | **25%** |
| > 40 mm | **1.49** | **0.72 to 3.1** | **13.56%** | **7.84%** |
| **Significant Prosthetic Valve Dysfunction** | | | | |
| **Diameter Group** | **HR** | **95%CI** | **PR** | **IR** |
| 35-40 mm | **Inf** | **Inf** | **2.29%** | **0** |
| > 40 mm | **0.61** | **0.11 to 3.46** | **1.69%** | **2.61%** |

CI = confidence interval; HR = hazard ratio

**Table S16. 5-Year Subdistribution Hazard of Stroke, Bleeding, NYHA Function Class II-IV, or Significant Aortic Valve Dysfunction among PR versus IR in the ‘Aorta Type’**

| **Stroke** | | | | |
| --- | --- | --- | --- | --- |
| **Diameter Group** | **HR** | **95%CI** | **PR** | **IR** |
| 45-52 mm | NA | NA | 0 | 0 |
| 52-60 mm | NA | NA | 0 | 0 |
| **Bleeding** | | | | |
| **Diameter Group** | **HR** | **95%CI** | **PR** | **IR** |
| 45-52 mm | 0.72 | 0.08 to 6.20 | 6.93% | 8.56% |
| 52-60 mm | 0 | 0 | 0 | 3.72% |
| **NYHA Function Class II-IV** | | | | |
| **Diameter Group** | **HR** | **95%CI** | **PR** | **IR** |
| 45-52 mm | 1.89 | 0.48 to 7.41 | 12.81% | 8.79% |
| 52-60 mm | 3.55 | 0.99 to 12.72 | 9.92% | 7.68% |
| **Significant Aortic Valve Dysfunction** | | | | |
| **Diameter Group** | **HR** | **95%CI** | **PR** | **IR** |
| 45-50 mm |  |  |  |  |
| 52-60 mm |  |  |  |  |

CI = confidence interval; HR = hazard ratio

**Table S17. 10-Year Subdistribution Hazard of Stroke, Bleeding, NYHA Function Class II-IV, or Significant Aortic Valve Dysfunction among PR versus IR in the ‘Aorta Type’**

| **Stroke** | | | | |
| --- | --- | --- | --- | --- |
| **Diameter Group** | **HR** | **95%CI** | **PR** | **IR** |
| 45-52 mm | NA | NA | 0 | 1.65% |
| 52-60 mm | NA | NA | 0 | 0 |
| **Bleeding** | | | | |
| **Diameter Group** | **HR** | **95%CI** | **PR** | **IR** |
| 45-52 mm | 0.72 | 0.08 to 6.20 | 6.93% | 10.21% |
| 52-60 mm | 0 | 0 | 0 | 5.59% |
| **NYHA function class II-IV** | | | | |
| **Diameter Group** | **HR** | **95%CI** | **PR** | **IR** |
| 45-52 mm | 1.89 | 0.48 to 7.41 | 19.21% | 10.44% |
| 52-60 mm | 3.55 | 0.99 to 12.72 | 21.83% | 7.68% |
| **Significant Aortic Valve Dysfunction** | | | | |
| **Diameter Group** | **HR** | **95%CI** | **PR** | **IR** |
| 45-52 mm |  |  |  |  |
| 52-60 mm |  |  |  |  |

CI = confidence interval; HR = hazard ratio

**Table S18. Between-Group 30-Day Freedom of the Cumulative Incidence of Death, Reoperation for Complications, Nonelective Cardiovascular Surgery for Adverse Events and Deep Wound Infection for PR and IR**

| **Valve Type** | | | | | |
| --- | --- | --- | --- | --- | --- |
| **Diameter Group** | **OR** | **95%CI** | **P Value** | **PR** | **IR** |
| 35-40 mm | Inf | Inf | 0.48 | 2.36% | 0 |
| > 40 mm | 0.60 | 0.19 to 1.93 | 0.39 | 3.50% | 5.67% |
| **Aorta Type** | | | | | |
| **Diameter Group** | **OR** | **95%CI** | **P Value** | **PR** | **IR** |
| 45-52 mm | 2.15 | 0.18 to 26.3 | 0.54 | 6.93% | 3.34% |
| 52-60 mm | 0 | 0 | 0.51 | 0 | 1.85% |

CI = confidence interval; OR = odds ratio
